# 2002–2017 anthropogenic emissions data for air quality modeling over the United States

**DOI:** 10.1016/j.dib.2023.109022

**Published:** 2023-03-02

**Authors:** Kristen M. Foley, George A. Pouliot, Alison Eyth, Michael F. Aldridge, Christine Allen, K. Wyat Appel, Jesse O. Bash, Megan Beardsley, James Beidler, David Choi, Caroline Farkas, Robert C. Gilliam, Janice Godfrey, Barron H. Henderson, Christian Hogrefe, Shannon N. Koplitz, Rich Mason, Rohit Mathur, Chris Misenis, Norm Possiel, Havala O.T. Pye, Lara Reynolds, Matthew Roark, Sarah Roberts, Donna B. Schwede, Karl M. Seltzer, Darrell Sonntag, Kevin Talgo, Claudia Toro, Jeff Vukovich, Jia Xing, Elizabeth Adams

**Affiliations:** a US Environmental Protection Agency, 109 T.W. Alexander Drive, Research Triangle Park, NC 27711, United States; b General Dynamics Information Technology, 79 T.W. Alexander Drive, Research Triangle Park, NC 27709, United States; c School of Environment, State Key Joint Laboratory of Environment Simulation and Pollution Control, Tsinghua University, Beijing, China; d University of North Carolina, Institute for the Environment, 100 Europa Drive, Suite 490, CB #1105, Chapel Hill, NC 27599, United States

**Keywords:** Emissions inventory, Emissions trends, Air quality modeling, CMAQ, SMOKE, MOVES

## Abstract

The United States Environmental Protection Agency (US EPA) has developed a set of annual North American emissions data for multiple air pollutants across 18 broad source categories for 2002 through 2017. The sixteen new annual emissions inventories were developed using consistent input data and methods across all years. When a consistent method or tool was not available for a source category, emissions were estimated by scaling data from the EPA's 2017 National Emissions Inventory with scaling factors based on activity data and/or emissions control information. The emissions datasets are designed to support regional air quality modeling for a wide variety of human health and ecological applications. The data were developed to support simulations of the EPA's Community Multiscale Air Quality model but can also be used by other regional scale air quality models. The emissions data are one component of EPA's Air Quality Time Series Project which also includes air quality modeling inputs (meteorology, initial conditions, boundary conditions) and outputs (e.g., ozone, PM_2.5_ and constituent species, wet and dry deposition) for the Conterminous US at a 12 km horizontal grid spacing.

**Specifications Table**

**Table:** 

Subject	Environmental Science: Air Pollution or Atmospheric Science
Specific subject area	Sixteen years of anthropogenic emission inputs for regional scale air quality modeling.
Type of data	Table Figure ASCII Binary (NetCDF file format)
How data were acquired	The data were generated using software tools.
Data format	Raw Analyzed
Description of data collection	The data include inputs and configuration information for an emissions processing system that can be used to generate inputs to an air quality model.
Data source location	Institution: U.S. Environmental Protection Agency City/Town/Region: Research Triangle Park NC Country: USA
Data accessibility	Repository name: Community Modeling and Analysis System (CMAS) Center Data Repository (Part of the University of North Carolina – Chapel Hill Dataverse Repository) Data identification number: Direct URL to data: https://doi.org/10.15139/S3/MW9OLB

## Value of the Data

• This dataset provides a consistent set of emissions data for emissions trends analysis and for air quality modeling over the Conterminous US for 2002–2017.
• This dataset includes all necessary data files and configuration scripts needed to use the Sparse Matrix Operator Kernel Emissions (SMOKE) processor to generate emissions input files for the Community Multiscale Air Quality (CMAQ) model. The emissions data can also be reformatted to support other air quality modeling systems.
• Air quality simulations using these emissions inventories can be used to study air quality impacts on human health and ecosystems over the last two decades. This multiyear dataset also supports dynamic and diagnostic model evaluation to inform improvements in the air quality modeling system.

## 1 Data Description

The Community Multiscale Air Quality modeling system (CMAQ; www.epa.gov/cmaq) estimates atmospheric concentrations for numerous chemicals, including ozone, PM_2.5_ and its constituents, and deposition of potentially harmful airborne chemical species. Decadal CMAQ simulations have been used for a diverse set of research and air quality management applications, including time-series-based epidemiological studies and nutrient loading assessments. Multiyear simulations have also been used for dynamic and diagnostic evaluation to increase confidence in the use of the air quality modeling system for estimating pollution trends. A key component of long-term air quality simulations is the development of anthropogenic emissions estimates that reflect intra- and interannual variabilty driven by changes in meterology, regulatory actions (e.g., motor vehicle emissions and fuel standards), and economic factors (e.g., changes in energy pricing for coal versus natural gas). This dataset was developed to provide a consistent set of long-term emissions inputs for human health, ecological, and evaluation applications of the CMAQ system or other air quality models.

**Fig. 1:**
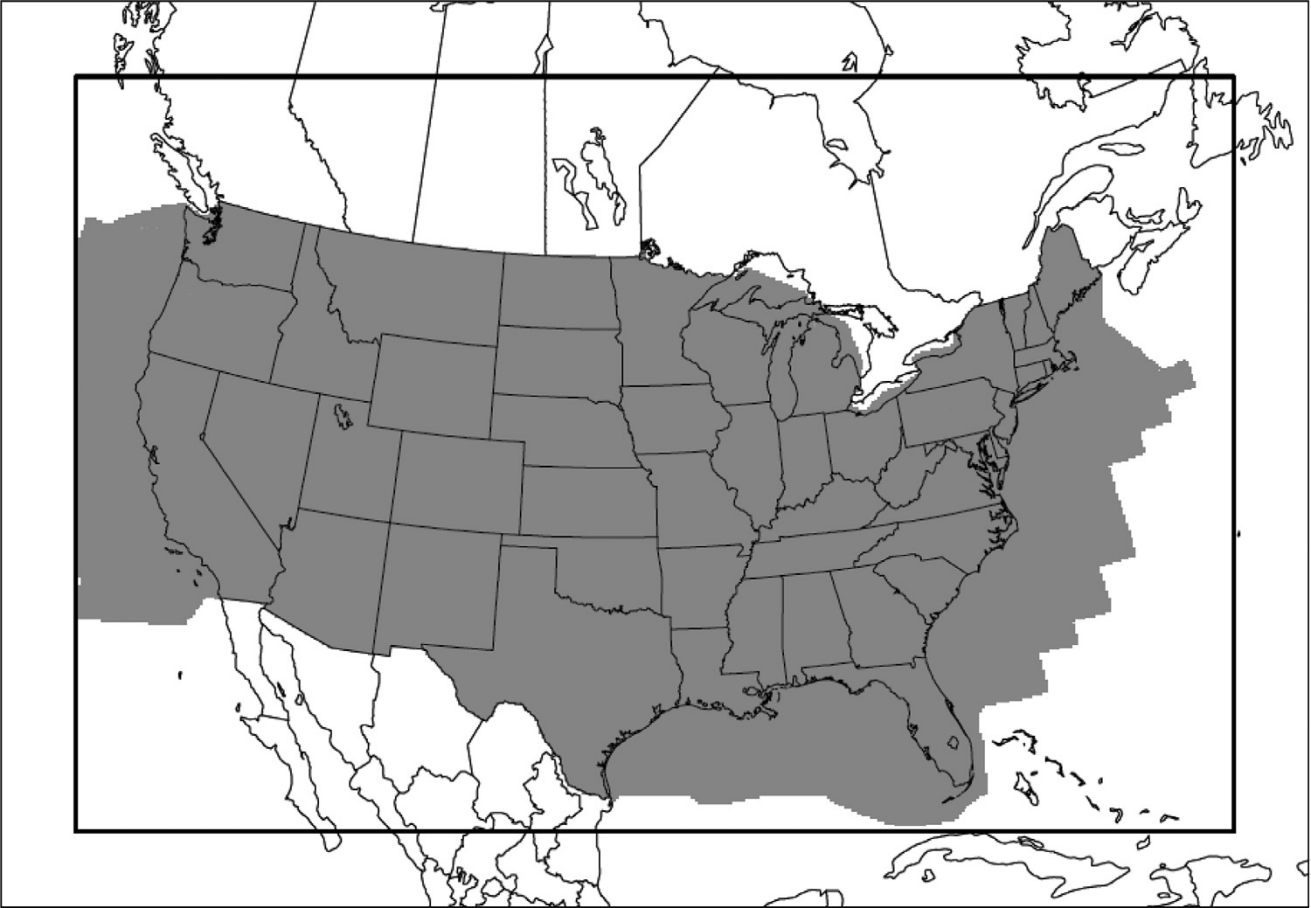
EQUATES modeling domain (“12US1”; 459 columns × 299 rows × 35 vertical layers) for the CONUS at a 12 km horizontal grid spacing is shown as the bold rectangle. The grey shading indicates grid cells that are considered in the CONUS, including federal waters, when calculating the emissions totals in Table 3.

This dataset includes annual emissions estimates for 2002-2017. The emissions data are one component of EPA's Air QUAlity TimE Series (EQUATES; www.epa.gov/cmaq/EQUATES) Project which also includes meteorology and air quality modeling of Conterminous US (CONUS) at 12 km horizontal grid spacing. Fig. 1 shows the modeling domain, henceforth referred to as 12US1. The emissions data were developed using, to the extent possible, consistent input data and methods across all years. This approach was taken to avoid artificial step-changes in emissions and air quality estimates due to changes in methodology that evolved over the sixteen year period that do not reflect real-world phenomena.

An example of the improved temporal consistency in EQUATES is the estimation of oil and gas emissions. Oil and gas emissions were not modeled as a separate category in EPA's 2002, 2005, and 2008 emissions modeling platforms. In addition, the oil and gas sources in EPA's National Emissions Inventories (NEI) were missing or underestimated for many states [1]. In contrast, oil and gas emissions for EQUATES were estimated using the same tool for all years and provide a more realistic representation of trends. Similarly, emissions from onroad vehicles were estimated in EQUATES using a single version of EPA's Motor Vehicle Emission Simulator (MOVES; https://www.epa.gov/moves) software. Previous emissions inventories used different versions of MOVES for different years. Updates across MOVES versions included changes in assumptions on fuel composition and vehicle operation (e.g., emissions from starts, hoteling, off network idling) which resulted in substantial increases or decreases in emissions estimates in some locations. A third example of a source with improved consistency in the EQUATES emissions over previous inventories is the volatile chemical products (VCP) sector. Methods for estimating VCP emissions have changed across the triannual NEIs from 2002 to 2017. As a result VCP emissions trends from older EPA inventories reflected both changes in activities that impact VCPs (e.g., an increase in the use of personal care products, or a decrease in the use of pesticides) and changes in estimation methods (e.g., the use of different data sources for estimating sales of personal care products or pesticides). EQUATES uses the VCPy framework for all years to estimates emissions based on chemical product usage, composition, and use timescale, and the characteristic evaporation timescale of the chemical product components [2]. Section 2 describes the EQUATES emission estimation methods used across all source categories including if/how the methods differ from previous EPA inventories. The remainder of this section describes emissions monthly and annual summary datasets and emissions inventory files that are available to download and provides a comparsion of emission trends from EQUATES and two other EPA sources for emissions data.

Emissions summary files listed in Table 1 are publicly available through the CMAS Center Dataverse Repository (https://doi.org/10.15139/S3/MW9OLB). Two versions of the annual emissions totals for 2002-2017 are provided: intentory (INV) emissions totals and model ready (MR) emissions totals. The differences between the INV and MR totals are described below. The Air Pollution Emissions Trends data (last row in Table 1) are not part of the EQUATES project but was used in the emissions comparison Figures in this section and is provided as a reference dataset.

**Table 1:** Description of emissions summary data files.

File Name (Size)	File Format (Size)	Description
README_EQUATES_v1.0_emissions_summaries.txt	ASCII	Metadata file describing the data contained in the emissions summary files
EQUATESv1.0_INV_emissions_annual_totals_by_pollutant.csv	Zipped ASCII file (1.5 KB)	2002-2017 annual total emissions for the lower 48 United States and District of Columbia (not including offshore sources) for 8 pollutants based on the INV files (see Table 2)
EQUATESv1.0_MR_emissions_annual_totals_by_pollutant.csv	Zipped ASCII file (1.8 KB)	2002-2017 annual total emissions summed over all grid cells overlapping CONUS including federal waters for 10 pollutants based on the MR files (see Table 3)
EQUATESv1.0_MR_emissions_annual_totals_by_source_and_pollutant.csv	Zipped ASCII file (28 KB)	2002-2017 annual total emissions by source category summed over all grid cells overlapping CONUS including federal waters for 10 pollutants based on the MR files (see Table 3)
EQUATES_CMAQ_12US1_grid_coordinates.csv.gz	Zipped ASCII file (5.4 MB)	CMAQ 12US1 grid information including row/column, Lambert conformal projected x/y coordinates for grid cell centers, longitude/latitude for the lower left and upper right corner of each grid cell, and the mask used for calculating CONUS-wide emissions totals
EQUATESv1.0_<pollutant>_12US1_annual_emissions_2002-2017.csv.gz	Zipped ASCII files (17 MB per file)	2002-2017 annual total gridded emissions for 9 pollutants (NOX, SO2, CO, PM2.5, OC, EC, VOC_regulatory, NMOG, NH3) for the 12US1 domain for 2002-2017 based on the MR files
EQUATESv1.0_<pollutant>_12US1_monthly_emissions_2002-2017.csv.gz	Zipped ASCII file (190 MB per file)	2002-2017 monthly total gridded emissions for 9 pollutants (NOX, SO2, CO, PM2.5, OC, EC, VOC_regulatory, NMOG, NH3) for the 12US1 domain for 2002-2017 based on the MR files
Emissions_comparison_EQUATES_vs_EPA_Trends_version_20220210.csv	ASCII file (108 KB)	Data from EQUATES, previous emissions modeling platforms, and EPA's Air Pollution Emissions Trends data used in the emissions comparison plots

In addition to the emission summary files in Table 1, the full set of INV emission data are provided to support their use in air quality modeling applications. The INV data are a set of emissions and meteorology files suitable for input into the Sparse Matrix Operator Kernel Emissions (SMOKE; https://www.cmascenter.org/smoke/) processor. Example scripts for using SMOKE to process INV total annual emissions data into the hourly gridded data in the MR files are provided in the INV packages, except for the inventories for Canada and Mexico. The scripts include SMOKE processing for temporal allocation, spatial allocation, and pollutant speciation. Meteorology data included in the INV files can be used in SMOKE to apply day-specific meteorology adjustments, e.g., fugitive dust emissions are adjusted based on soil moisture, soil type, and snow cover.

The INV emissions and meteorology files are accompanied by ancillary input files needed for SMOKE processing and example scripts showing how to configure and run SMOKE. The emissions data are ASCII files and the meteorology files are compressed NetCDF4 format [3] due to the large size of the datasets. Files are consolidated into tar files using a Unix-based utility to package files together and compressed into .GZ files with GNU Zip compression for easier download. Table S1 describes the tar packages and how the files are organized for each year. The INV files do not include biogenic or geogenic sources that are calculated online by CMAQ: biogenic emissions (including nitric oxide from soil and volatile organic compounds from vegetation), sea spray, windblown dust, and nitrogen oxides from lightning.

After the INV files are processed through SMOKE they generate MR emission data either as gridded or point (sometimes referred to as “inline”) file structures suitable for input to the CMAQ model. The sixteen years of MR emissions data are over 5TB in size and are not included in the DataVerse data repository for this paper. However, several sets of MR emissions summary files are provided to support emissions trend analysis and to facilitate diagnostic evaluation of air quality model simulations. The MR emissions summaries include annual CONUS totals by source category and summed across all sources as well as gridded annual and monthly totals. The gridded annual and monthly MR emissions summaries cover the conterminous US utilizing 12 km horizontal grid spacing on a Lambert-conformal projection (Fig. 1). Because the modeling domain covers northern Mexico and a large portion of southern Canada, the gridded MR summary files include Canada and Mexico emissions for those regions, although the underlying inventories for these countries are not part of the INV format files. Additional information on the MR emissions data, including how to access the full set of CMAQ inputs, is available from the EQUATES website.

Table 2 provides annual total CONUS emissions in short tons based on the INV emissions files for nitrogen oxides (NO_X_ ), sulfur dioxide (SO_2_), carbon monoxide (CO), particulate matter with diameter 2.5 µm and smaller (PM_2.5_), particulate matter with diameter 10 µm and smaller (PM_10_), regulatory volatile organic compounds (VOC, defined as in the Code of Federal Regulations, 40 CFR 51.100), ammonia (NH_3_) excluding emissions from fertilizer, and NH_3_ emissions from fertilizer. NH_3_ emissions from fertilizer are separated because they were calculated online in CMAQ and then the model output is reformatted as an emissions inventory file. Emissions totals are for the lower 48 states and the District of Columbia (Lower 48) and do not include emissions from offshore sources, although these sources are included in the actual INV emissions files. Offshore sources include emissions from offshore oil and gas drilling in the Gulf of Mexico and emissions from commercial marine vessels away from the shoreline. Port emissions and emissions from commercial marine vessels within a buffer of 3 to 10 miles from the shore are included in the emissions totals. Offshore sources are excluded in the annual total summaries to support the emissions comparison in this section.

**Table 2:** Annual total (10^3^ short tons) inventory (INV) emissions for the Lower 48 for NO_X_, SO_2_, CO, PM_2.5_, PM_10_, regulatory VOC, NH_3_ excluding emissions from fertilizer, NH_3_ emissions from fertilizer (calculated online by CMAQ).

Year	NO_X_	SO_2_	CO	PM_2.5_	PM_10_	Regulatory VOC	NH_3_ (no fertilizer)	NH_3_ from fertilizer
2002	24,922	14,752	97,137	5,048	18,323	17,655	3,046	1,277
2003	24,602	14,864	98,017	5,547	18,977	18,565	3,130	1,281
2004	22,341	14,506	86,397	5,156	18,559	17,024	3,033	1,244
2005	21,530	14,477	83,635	5,351	18,871	17,105	3,082	1,524
2006	20,021	12,368	80,161	5,381	18,772	16,912	3,110	1,394
2007	18,745	11,524	77,160	5,375	18,812	17,028	3,196	1,570
2008	17,956	11,418	68,746	5,114	18,499	15,791	3,175	1,406
2009	15,324	7,954	62,679	4,684	17,946	14,179	3,085	1,239
2010	15,061	6,918	58,739	4,689	17,944	13,859	3,028	1,389
2011	14,364	6,314	60,933	5,040	18,323	14,933	3,113	1,425
2012	13,479	5,024	57,890	4,794	18,076	14,979	3,090	1,439
2013	12,650	4,773	53,402	4,496	17,763	13,554	3,013	1,410
2014	11,936	4,525	52,593	4,544	17,835	13,690	2,970	1,349
2015	10,869	3,438	54,458	4,741	18,229	14,353	3,075	1,439
2016	9,724	2,575	49,311	4,571	18,202	12,997	3,088	1,423
2017	9,316	2,469	59,794	5,634	19,636	15,735	3,317	1,395

Table 3 provides the emissions totals based on the MR emissions files for NO_X_, SO_2_, CO, PM_2.5_, primary organic carbon (POC), primary elemental carbon (PEC), VOC, non-methane organic gases (NMOG), NH_3_ excluding emissions from fertilizer, and NH_3_ emissions from fertilizer. The emissions totals in Table 2 differ from those in Table 3 for several reasons related to the SMOKE processing that creates the model-ready emissions. For example, SMOKE processing of the emissions from electrical generating units includes replacing NO_X_ and SO_2_ emissions reported in EPA's triannual NEIs with hourly unit-specific Continuous Emission Monitoring data. In addition, the processing of emissions through SMOKE adjusts the emissions from fugitive dust to account for decreased dust following precipitation events and deposition of dust on near-source vegetation. This post-processing step decreases total annual PM_2.5_ emissions from all sources on the order of 20-25%. The temporal allocation step in SMOKE allocates annual total emissions to hourly emissions using month-of-year, day-of-week (accounting for weekdays, weekends, and holidays), and diurnal profiles. The annual totals after the temporal allocation process are slightly different than the pre-SMOKE inventory totals.

**Table 3:** Annual total (10^3^ short tons) model-ready (MR) emissions over all grid cells overlapping the CONUS (including federal waters) as shown in the map in Fig. 1 for NO_X_, SO_2_, CO, PM_2.5_, POC, PEC, regulatory VOC, NMOG, NH_3_ excluding emissions from fertilizer, NH_3_ from fertilizer (calculated online by CMAQ).

Year	NO_X_	SO_2_	CO	PM_2.5_	POC	PEC	Regulatory VOC	NMOG	NH_3_ (no fertilizer)	NH_3_ from fertilizer
2002	25,196	15,038	96,287	3,776	969	463	17,582	19,414	2,943	1,282
2003	24,607	15,594	97,728	4,326	1,225	481	18,605	20,534	3,134	1,281
2004	22,817	14,902	86,377	3,916	1,034	458	17,135	19,043	3,038	1,246
2005	21,862	14,841	83,603	4,155	1,127	456	17,192	19,170	3,087	1,516
2006	20,345	12,717	80,136	4,115	1,182	444	17,014	19,072	3,116	1,394
2007	19,066	11,867	76,897	4,152	1,263	410	17,075	19,161	3,202	1,561
2008	17,991	10,425	68,792	3,899	1,160	385	15,923	18,043	3,185	1,408
2009	15,601	8,224	62,644	3,446	1,036	346	14,271	16,196	3,092	1,244
2010	15,388	7,051	58,744	3,474	1,051	351	13,949	15,857	3,034	1,392
2011	14,689	6,527	60,931	3,840	1,231	337	15,017	17,037	3,120	1,430
2012	13,824	5,257	57,914	3,677	1,138	311	15,090	17,235	3,100	1,443
2013	12,982	5,007	53,432	3,267	1,009	290	13,649	15,591	3,020	1,414
2014	12,236	4,740	52,601	3,318	1,034	276	13,776	15,804	2,976	1,354
2015	11,169	3,428	54,444	3,503	1,143	264	14,432	16,473	3,083	1,439
2016	10,049	2,583	49,355	3,347	1,066	238	13,104	14,997	3,100	1,424
2017	9,636	2,481	59,803	4,404	1,571	260	15,808	17,820	3,324	1,395

The SMOKE system uses source-specific speciation profiles [4,5] to map species to the chemical mechanism used in the EQUATES CMAQ simulations. Pollutant speciation is based on the Carbon Bond 6 version r3 (CB6r3) chemical mechanism [6] and CMAQ's AERO6 aerosol module. In addition to VOC emissions totals, Table 3 includes NMOG which better reflects the total organic gases emitted to air. The NMOG emissions are calculated, in units of short tons, as a sum of the following MR emissions species:

$$\begin{array}{ccc} \text{NMOG} & = & ACET+ALD2+ALDX+BENZ++ETH+ETHA+ETHY+\;ETOH+FORM+IOLE\\ & & +\;ISOP+KET+MEOH+NAPH+NVOL+OLE+PAR+PRPA+TERP+TOL\\ & & +\;UNR+XYLMN\; \end{array}$$

The definition for the model species (e.g., ACET, ALD2, ALDX) can be found in the CMAQ “cb6r3_ae7_aq” Species Table available from the CMAQ GitHub repository (https://github.com/USEPA/CMAQ/tree/5.3.2/CCTM/src/MECHS).

**Fig. 2:**
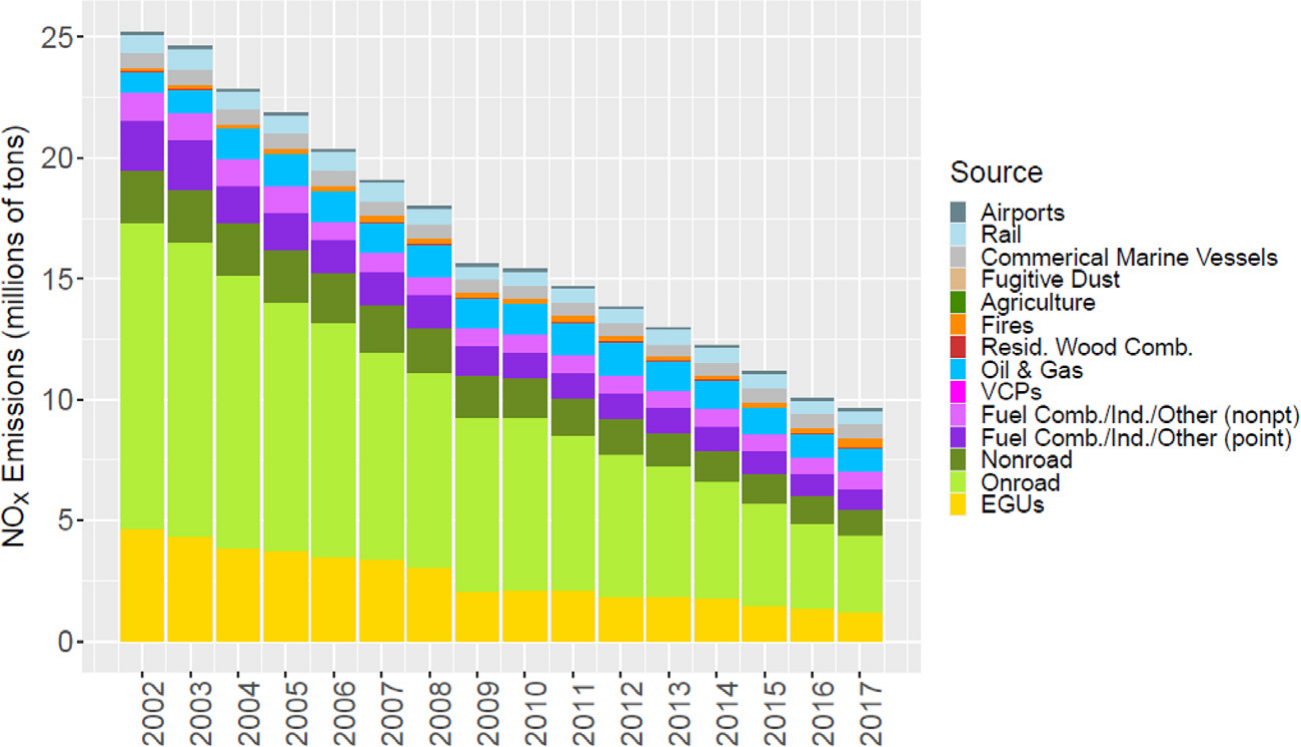
Annual total NO_X_ emissions (10^6^ short tons as NO_2_) by source over all grid cells overlapping the CONUS (including federal waters) from the MR emissions files. Note that NO_X_ from soil or lightning are not included as these are calculated online by CMAQ.

Figs. 2 through 10 show the annual total emissions from the MR emissions summary files grouped into 14 source categories: airports; rail; commercial marine vessels; fugitive dust; agriculture; fires (including agricultural fires, wildfires, and prescribed fires); residential wood combustion; oil and gas; volatile chemical products; fuel combustion, industrial and other nonpoint sources (referred to nonpt throughout the paper); fuel combustion, industrial and other point sources (referred to as ptnonipm throughout the paper); nonroad mobile; onroad mobile; electric generating utilities (EGUs).

**Fig. 3:**
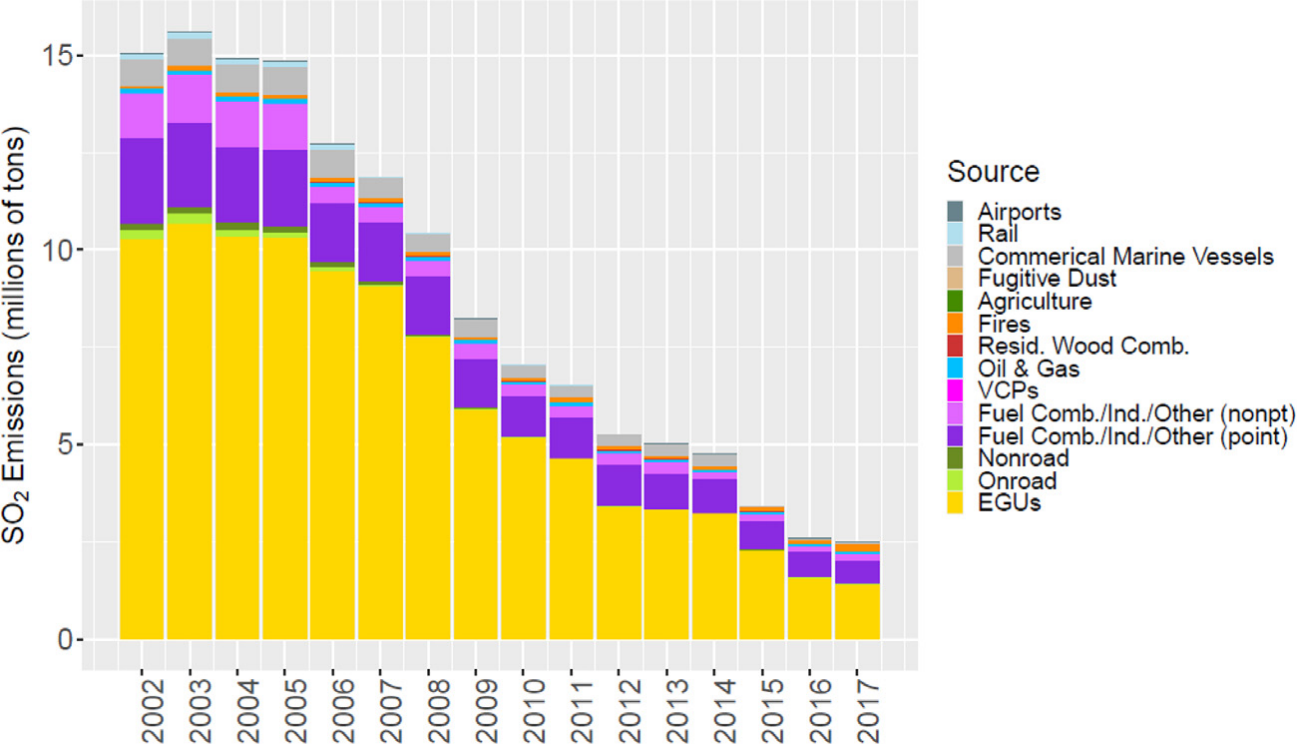
Annual total SO_2_ emissions (10^6^ short tons) by source over all grid cells overlapping the CONUS (including federal waters) from the MR emissions files.

**Fig. 4:**
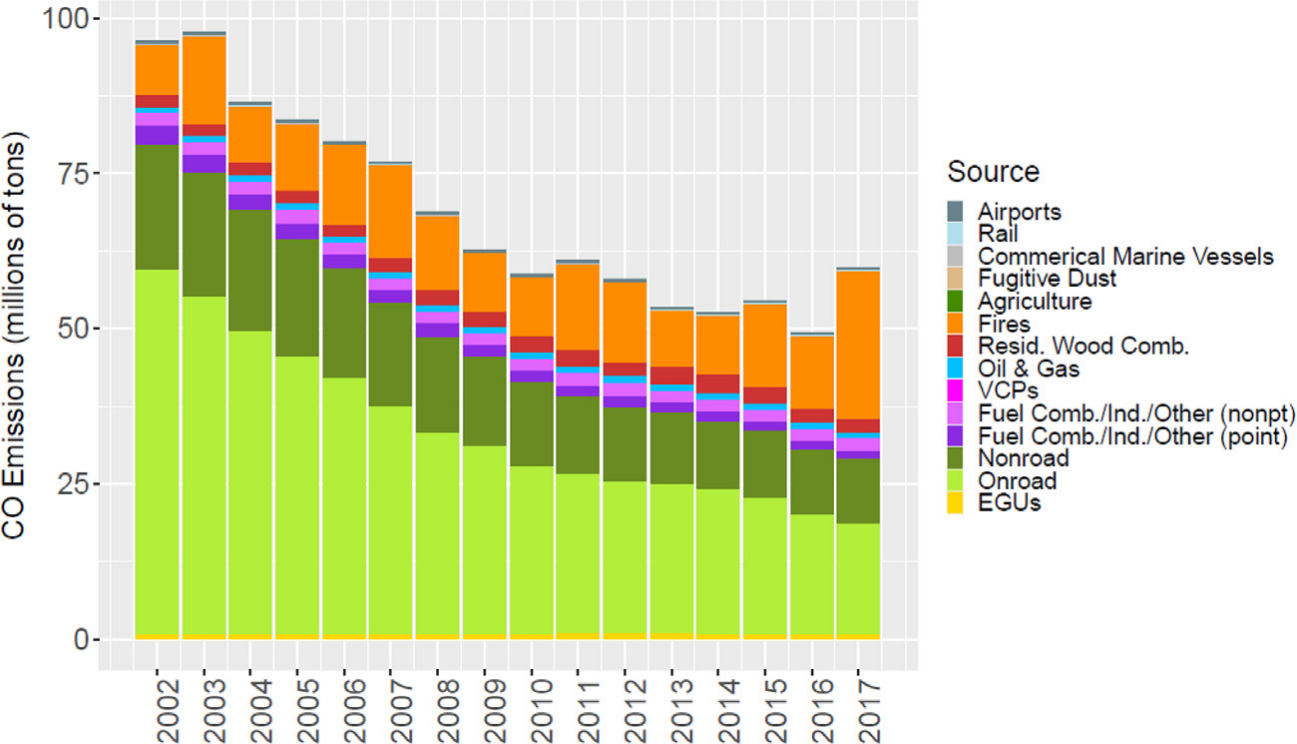
Annual total CO emissions (10^6^ short tons) by source over all grid cells overlapping the CONUS (including federal waters) from the MR emissions files. Note that emissions from biogenic sources are not included as these are calculated online by CMAQ.

**Fig. 5:**
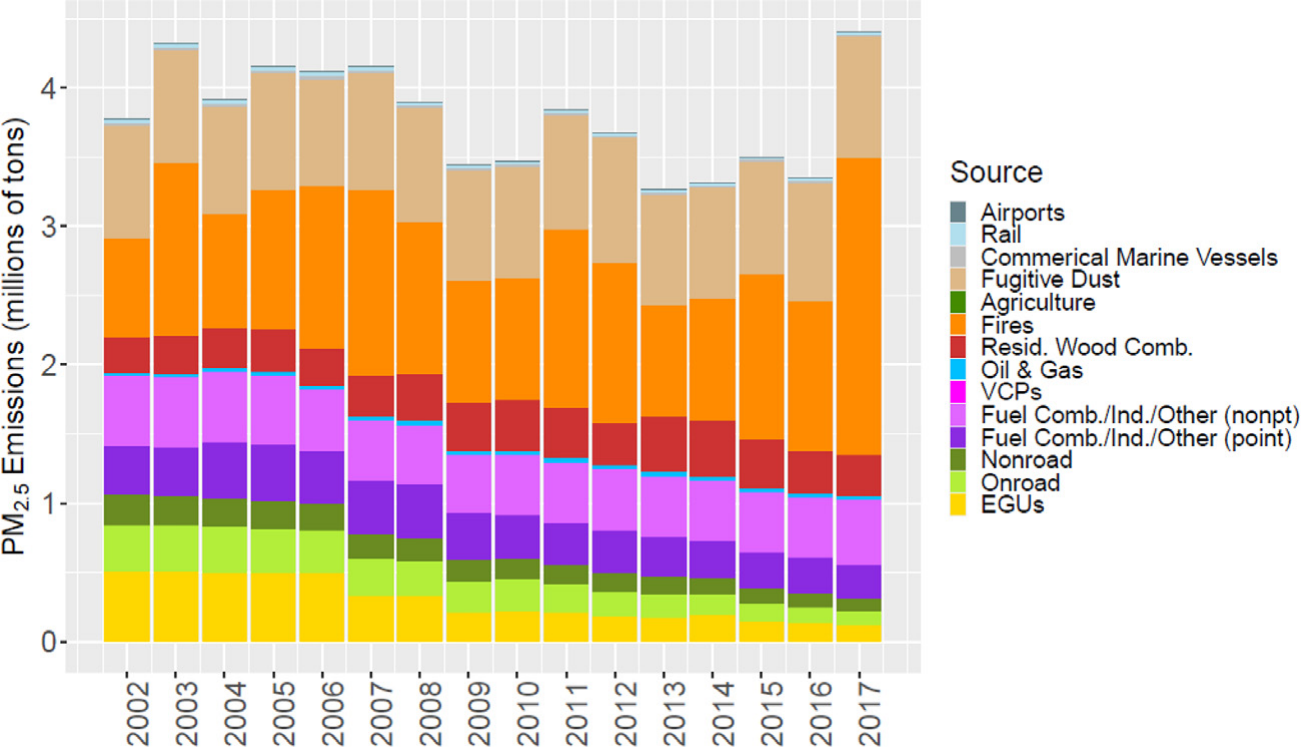
Annual total PM_2.5_ emissions (10^6^ short tons) by source over all grid cells overlapping the CONUS (including federal waters) from the MR emissions files. Note that emissions from sea spray are not included as these are calculated online by CMAQ.

**Fig. 6:**
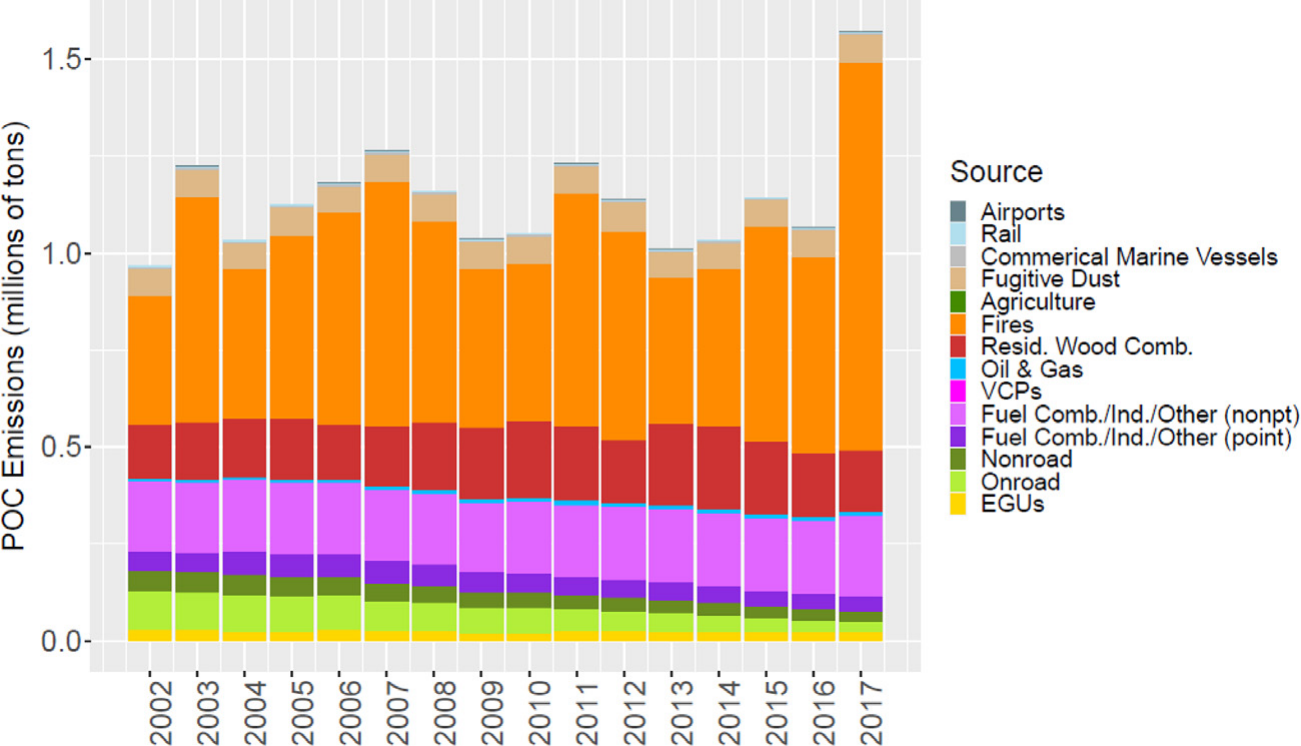
Annual total POC emissions (10^6^ short tons) by source over all grid cells overlapping the CONUS (including federal waters) from the MR emissions files.

**Fig. 7:**
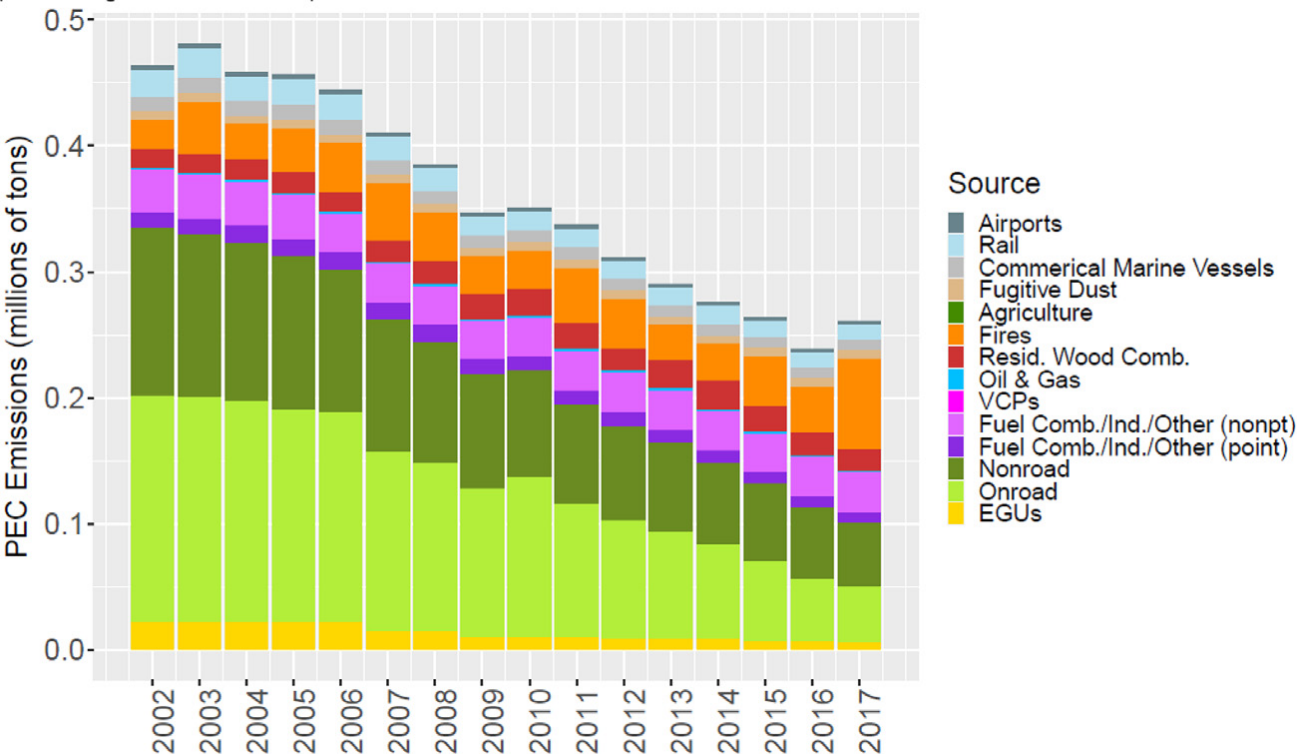
Annual total PEC emissions (10^6^ short tons) by source over all grid cells overlapping the CONUS (including federal waters) from the MR emissions files.

**Fig. 8:**
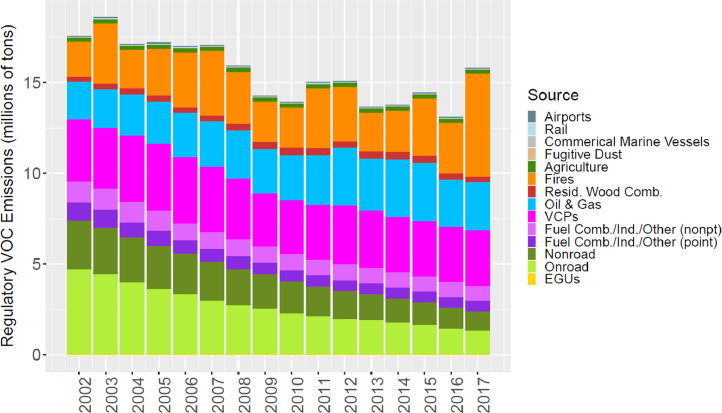
Annual total regulatory VOC emissions (10^6^ short tons) by source over all grid cells overlapping the CONUS (including federal waters) from the MR emissions files. For VCPs, VOC was determined from NMOG using the ratio of NMOG to regulatory VOCs of 1.18 following Seltzer et al. [2]. Note that emissions from biogenic sources are not included as these are calculated online by CMAQ.

**Fig. 9:**
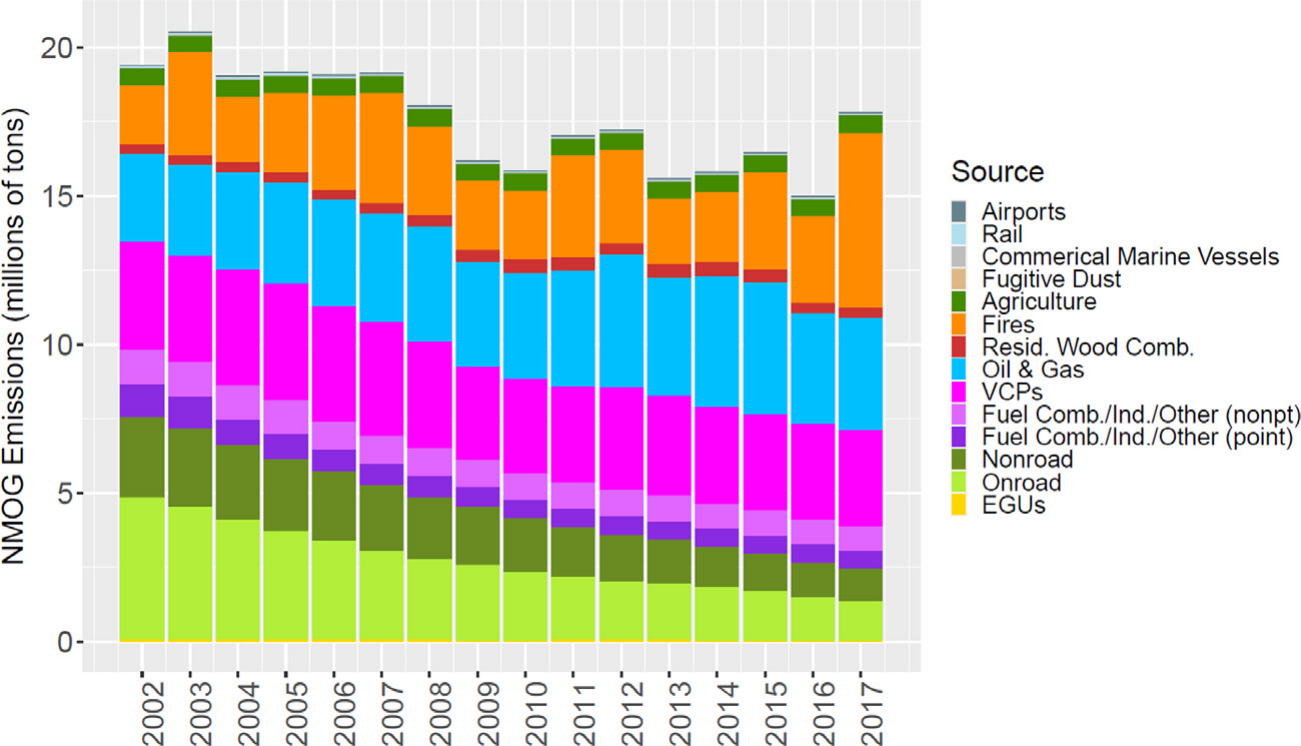
Annual total NMOG emissions (10^6^ short tons) by source over all grid cells overlapping the CONUS (including federal waters) from the MR emissions files. Note that emissions from biogenic sources are not included as these are calculated online by CMAQ.

**Fig. 10:**
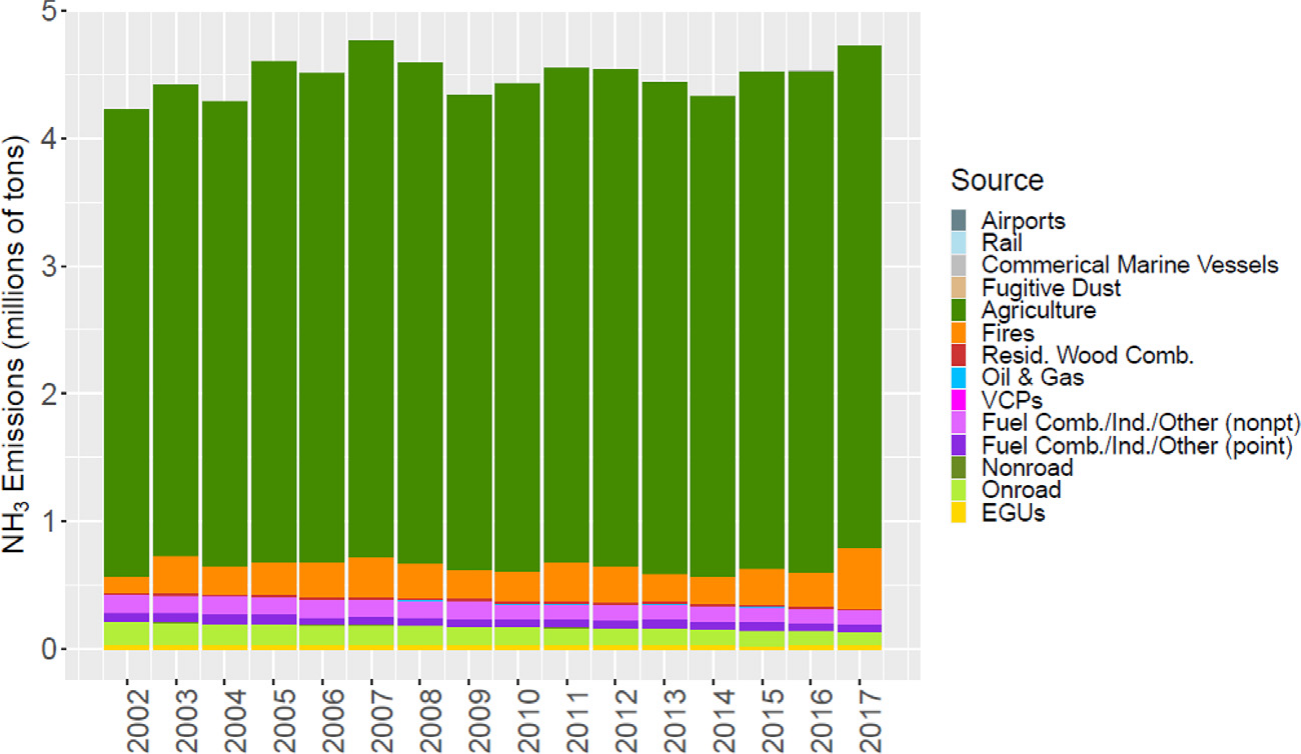
Annual total NH_3_ emissions (10^6^ short tons) by source over all grid cells overlapping the CONUS (including federal waters) from the MR emissions files. Note that emissions from agriculture (top of stacked bar and shown in green) include NH_3_ emissions from fertilizer that were calculated online by CMAQ.

These source categories are described in more detail in Section 2. The emissions totals in Tables 2 and 3 and the source category-specific totals displayed in the figures are available to download (see Table 1).

**Fig. 11:**
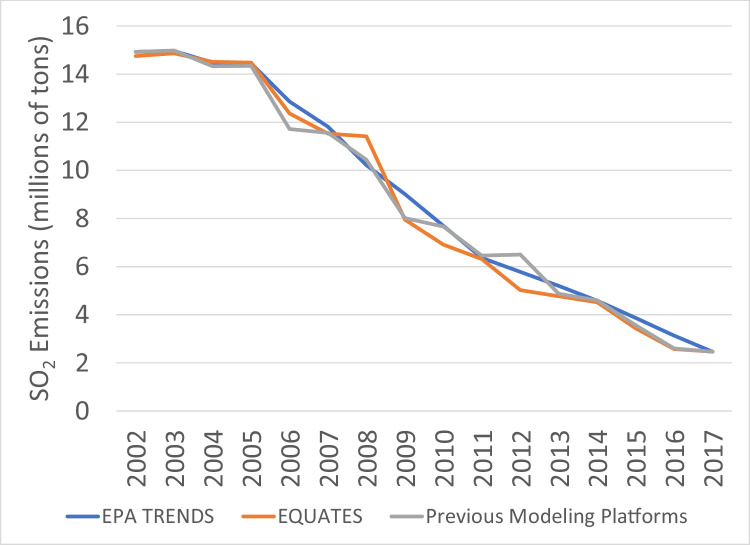
Annual total SO_2_ emissions (10^6^ short tons) over the Lower 48 from EQUATES INV files (orange), EPA Trends data (blue) and previous emissions modeling platform data (grey).

**Fig. 12:**
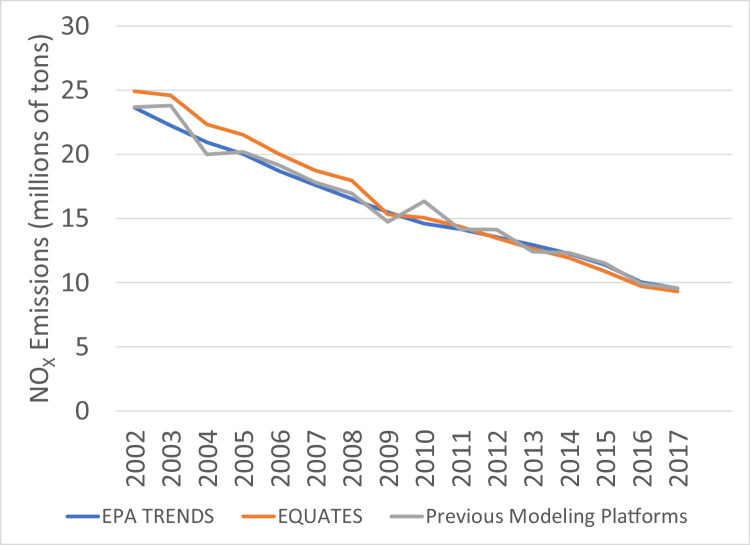
Annual total NO_x_ emissions (10^6^ short tons) over the Lower 48 from EQUATES INV files (orange), EPA Trends data (blue) and previous emissions modeling platform data (grey).

**Fig. 13:**
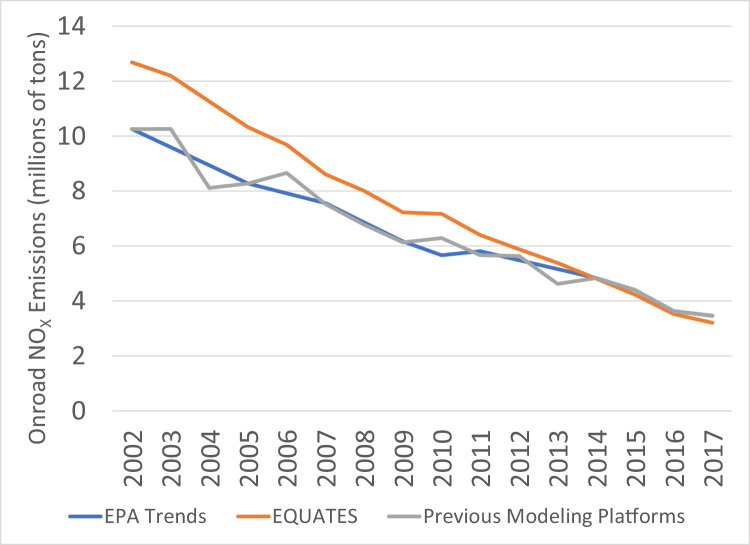
Annual total NO_x_ emissions from onroad vehicles (10^6^ short tons) over the Lower 48 from EQUATES INV files (orange), EPA Trends data (blue) and previous emissions modeling platform data (grey).

**Fig. 14:**
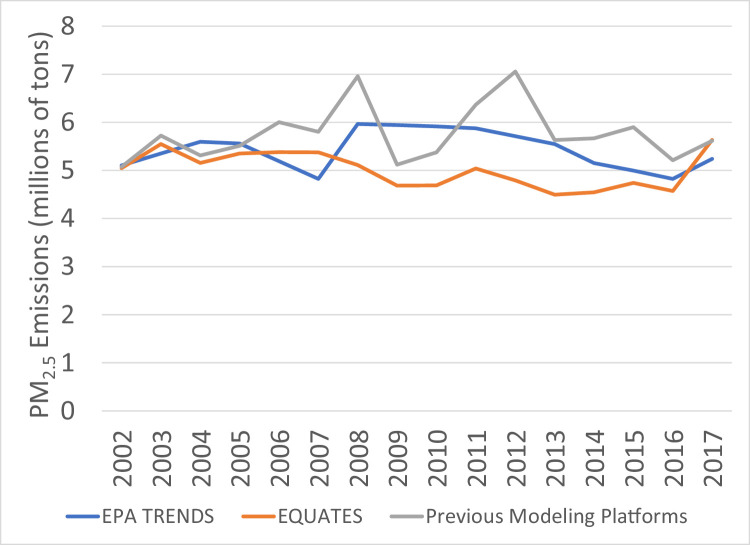
Annual total PM_2.5_ emissions (10^6^ short tons) over the Lower 48 from EQUATES INV files (orange), EPA Trends data (blue) and previous emissions modeling platform data (grey).

**Fig. 15:**
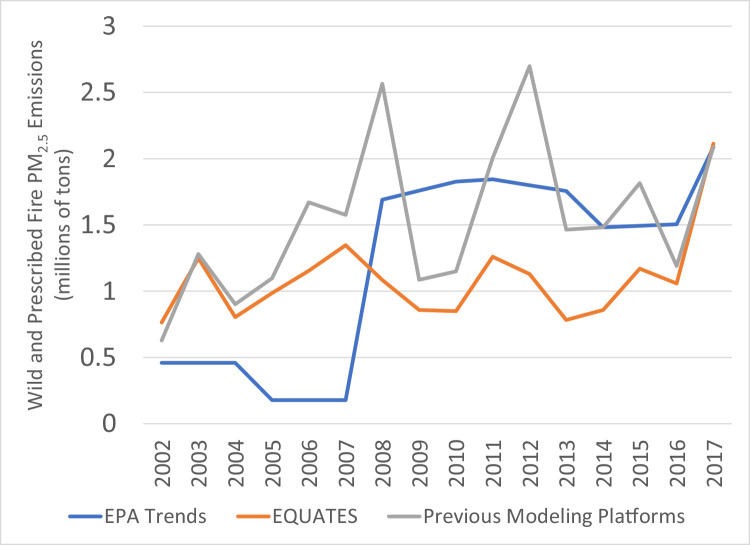
Annual total PM_2.5_ emissions from wild and prescribed fires (10^6^ short tons) over the Lower 48 from EQUATES INV files (orange), EPA Trends data (blue) and previous emissions modeling platform data (grey).

**Fig. 16:**
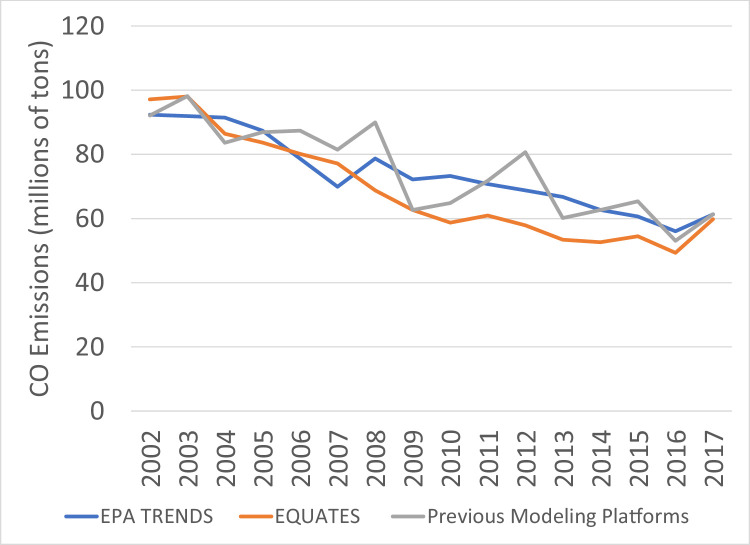
Annual total CO emissions (10^6^ short tons) over the Lower 48 from EQUATES INV files (orange), EPA Trends data (blue) and previous emissions modeling platform data (grey).

**Fig. 17:**
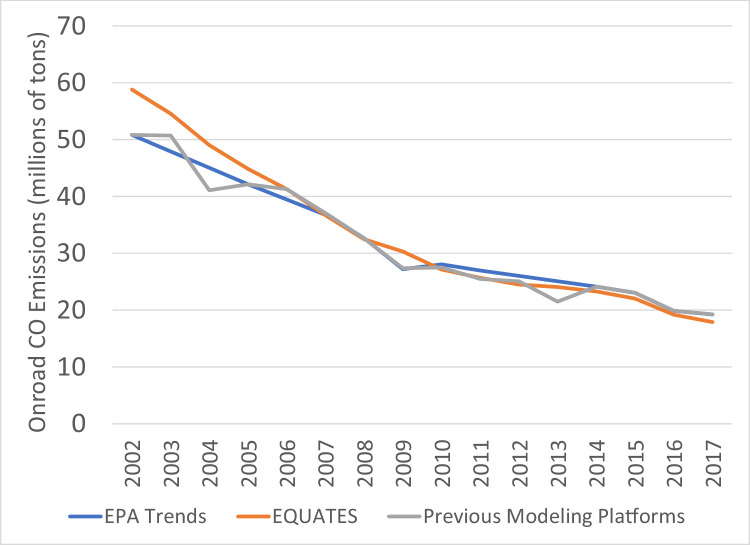
Annual total CO emissions from onroad vehicles (10^6^ short tons) over the Lower 48 from EQUATES INV files (orange), EPA Trends data (blue) and previous emissions modeling platform data (grey).

**Fig. 18:**
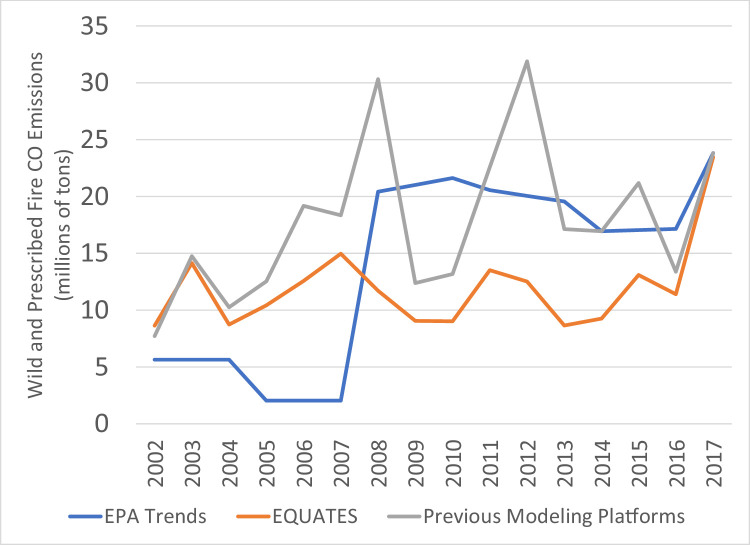
Annual total CO emissions from wild and prescribed fires (10^6^ short tons) over the Lower 48 from EQUATES INV files (orange), EPA Trends data (blue) and previous emissions modeling platform data (grey).

**Fig. 19:**
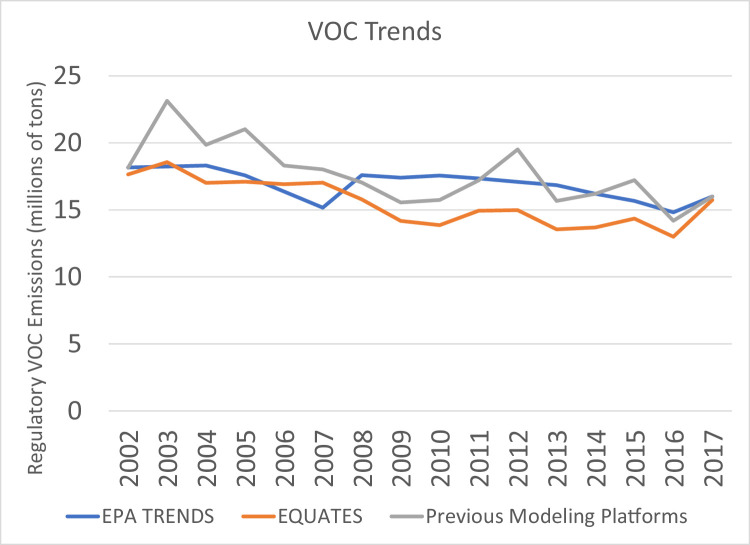
Annual total regulatory VOC emissions (10^6^ short tons) over the Lower 48 from EQUATES INV files (orange), EPA Trends data (blue) and previous emissions modeling platform data (grey).

**Fig. 20:**
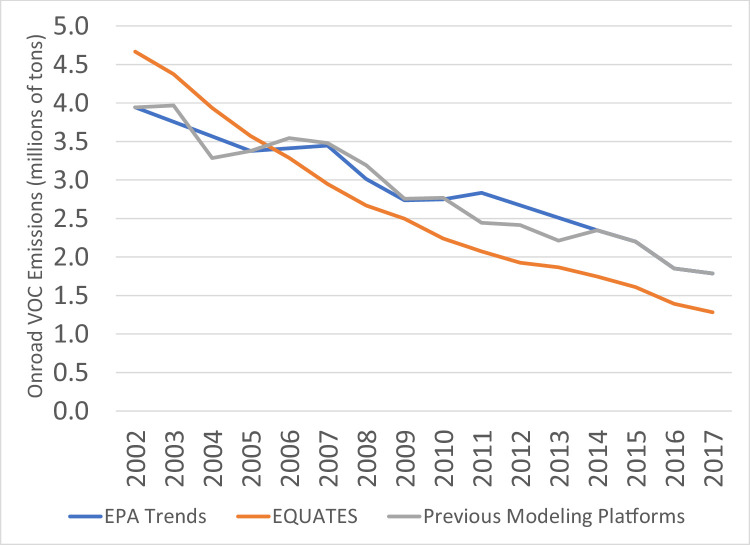
Annual total regulatory VOC emissions from onroad vehicles (10^6^ short tons) over the Lower 48 from EQUATES INV files (orange), EPA Trends data (blue) and previous emissions modeling platform data (grey).

**Fig. 21:**
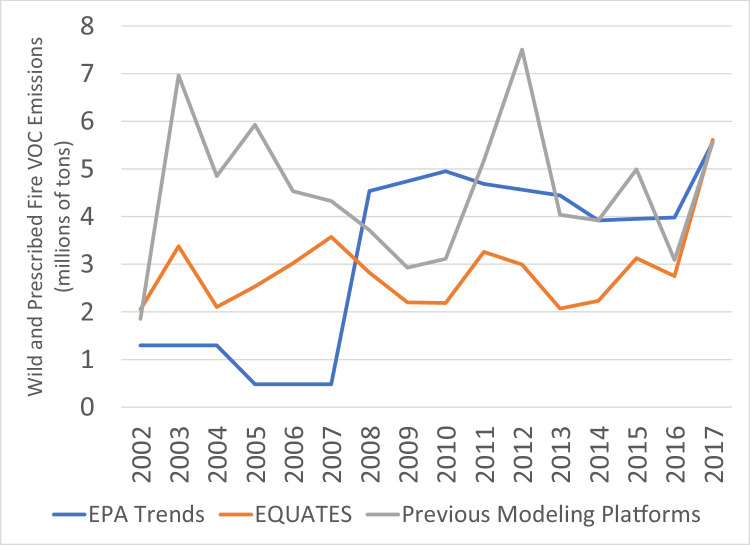
Annual total regulatory VOC emissions from wild and prescribed fires (10^6^ short tons) over the Lower 48 from EQUATES INV files (orange), EPA Trends data (blue) and previous emissions modeling platform data (grey).

**Fig. 22:**
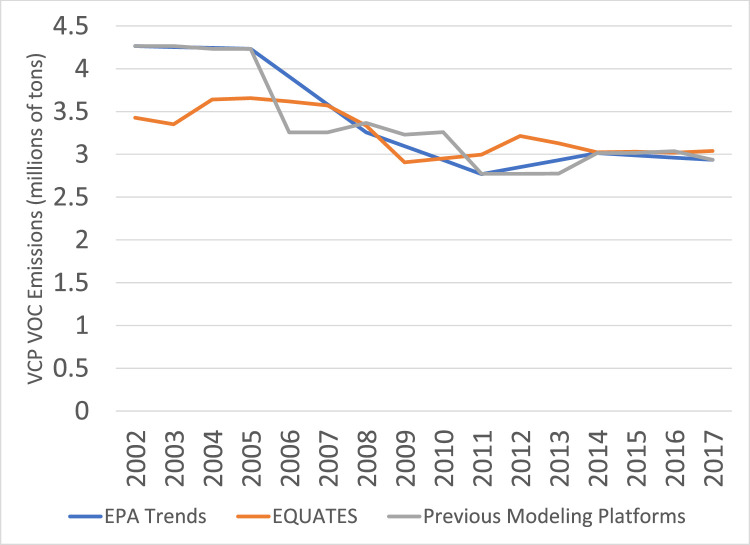
Annual total regulatory VOC emissions from VCPs (10^6^ short tons) over the Lower 48 from EQUATES INV files (orange), EPA Trends data (blue) and previous emissions modeling platform data (grey).

**Fig. 23:**
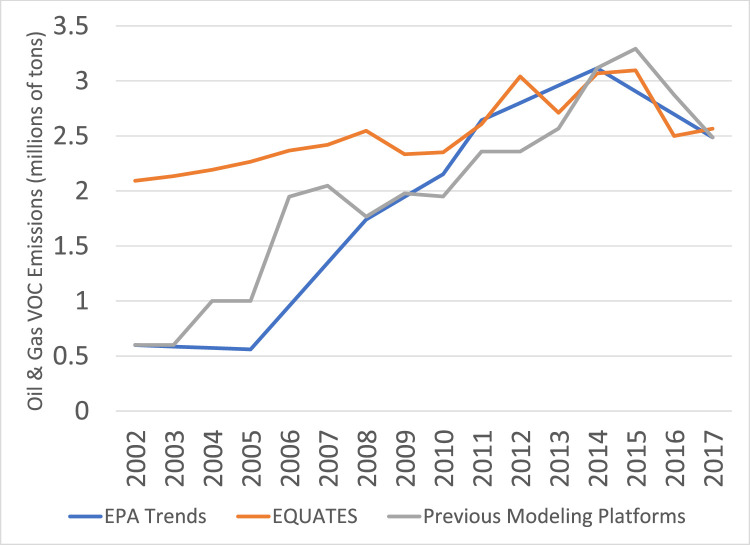
Annual total regulatory VOC emissions from oil and gas sources (10^6^ short tons) over the Lower 48 from EQUATES INV files (orange), EPA Trends data (blue) and previous emissions modeling platform data (grey).

**Fig. 24:**
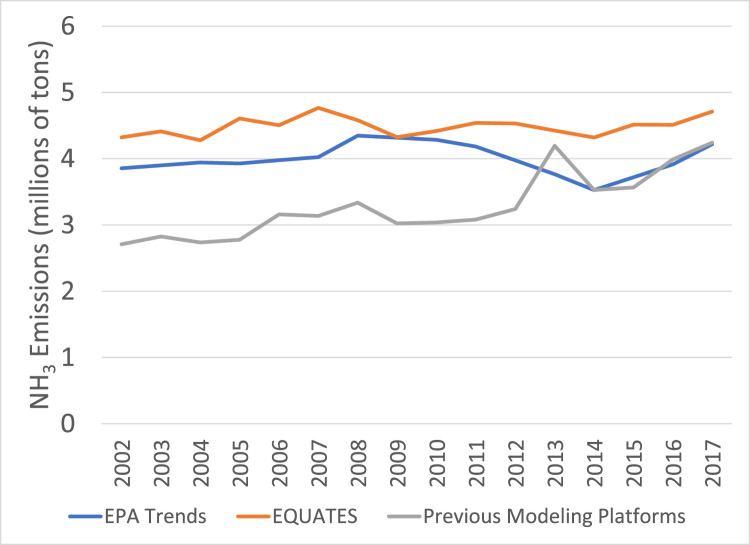
Annual total NH_3_ emissions (10^6^ short tons) over the Lower 48 from EQUATES INV files (orange), EPA Trends data (blue) and previous emissions modeling platform data (grey).

**Fig. 25:**
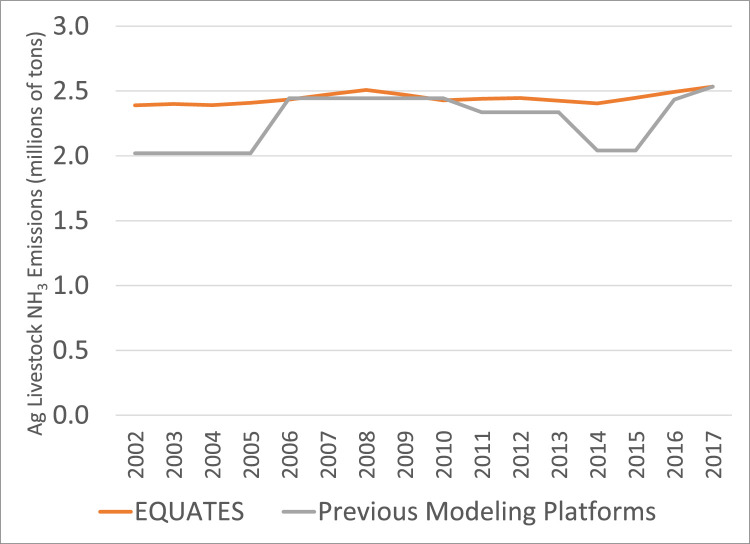
Annual total NH_3_ emissions from livestock, part of the agriculture category, (10^6^ short tons) over the Lower 48 from EQUATES INV files (orange) and previous emissions modeling platform data (grey).

The file “Emissions_comparison_EQUATES_vs_EPA_Trends_version_20220210.csv” provided in the EQUATES DataVerse repository allows for the comparison of annual total emissions from the EQUATES INV data to totals from previous EPA emissions modeling platforms (Tables S3, S4) and the EPA's Air Pollution Emissions Trends data (Table S2), henceforth referred to as EPA Trends data. Fig. 11, Fig. 12, Fig. 13, Fig. 14, Fig. 15, Fig. 16, Fig. 17, Fig. 18, Fig. 19, Fig. 20, Fig. 21, Fig. 22, Fig. 23, Fig. 24, Fig. 25 provide source specific comparisions of these three data sources to illustrate how the EQUATES methodology for particular sources differs from previous estimates.

The EPA Trends data for 2002–2017 are based on the triannual NEIs. Emissions values for non-NEI years are supplemented with year-specific data, held constant at the value of an adjacent NEI year, or interpolated between the nearest NEI years. The EPA Trends data are updated periodically with new data or methods. The emssions comparison data in this repository are based on the February 10, 2022 version of the EPA Trends data. State totals for the Lower 48 (48 states and the District of Columbia, not including offshore sources) were summed to create national totals comparable to the EQUATES INV data for SO_2_, NO_X_, PM_2.5_, CO, Regulatory VOC, and NH_3_. The EPA Trends data include 15 source categories which differ from the source categories used in the EQUATES data. The EQUATES annual summaries were recalculated to align with the EPA Trends categories to create an equivalent dataset for this comparison. Agricultural sources (fertilizer, livestock and agriculural fires) are included in the “MISCELLANEOUS” category of the EPA Trends data, however they are retained as separate categories in the EQUATES and previous modeling platform summaries which also have an “ALL OTHER SOURCES” category.

### 1.1 SO_2_ and NO_X_

For most pollutants, the EPA Trends data have less year-to-year variability than the other two emissions datasets due to the use of interpolation between triennial NEI years. In general, the EQUATES emissions trends are smoother than the totals from the previous modeling platforms, reflecting the use of newer and more consistent methods. Trends from the three data sets are most similar for SO_2_ and NO_X_ emissions (Figs. 11, 12). This similarity is expected since the emissions from EGUs, fuel combustion, and industrial sources make up a substantial portion of these pollutant emissions and the data sources and methods used for these categories are very similar or identical in the EQUATES inventories, EPA Trends, and previous modeling platforms (see Sections 2.1.2, 2.1.11, 2.1.12).

The EQUATES NO_X_ emissions for 2002–2008 are 5–11% higher than the EPA Trends totals. This difference is primarily due to higher onroad NO_X_ emissions in EQUATES (Fig. 13) from the use of the MOVES3 model for all years and consistent inputs (see Secton 2.1.8). Onroad emissions from previous modeling platforms used older versions of the mobile emissions model (MOVES2010b, MOVES2014a, MOVES2014b) and were based on older methods and input data. An example of this is the development of year-specific vehicle miles traveled (VMT) data. The EPA Trends data and the previous modeling platforms included state submitted VMT data which created inconsistencies across years and states in how VMT was being estimated. In addition, the methods used for estimating VMT when state-submitted data were not available have improved with each new NEI, allowing for more refined estimates but again creating inconsistencies or step-changes across years.

As described in Section 2.1.8, new VMT data were developed for EQUATES with the same methodology used for all states and years. The new VMT data were then used to derive other types of MOVES activity data: vehicle population (VPOP), hoteling, vehicle starts, off-network ideling hours (ONI). EQUATES new VMT is generally greater than the previous VMT estimates, which leads to higher emissions. Another cause for the differences in onroad NO_X_ emissions is due to a change in the estimated age distribution for combination trucks in MOVES3. In MOVES3 the age distribution of the combination trucks is shifted toward more older vehilces compared to earlier versions of MOVES, which leads to higher emissions.

### 1.2 PM_2.5_

While PM_2.5_ emissions for 2002 and 2017 are very similar across the three datasets, there are many differences in the intermediate years (Fig. 14). The previous modeling platforms show the greatest year-to-year variability with large peaks in 2008 and 2012. The EPA Trends data show a 24% increase from 2007 and 2008, while the EQUATES annual emissions are all within ±12% of 2002 levels. A large contributor to these discrepancies is a divergence in the estimation methods for emissions from prescribed and wildfires (Fig. 15). The EPA Trends data are missing emissions from prescribed fires in 2002-2007. Prescribed fires are added in 2008 and are then interpolated between NEI years (e.g., 2009 is an interpolation between 2008 and 2011). Wildfire emissions in the Trends data are not always year-specific but are based on previous NEIs in some years (e.g., 2003 and 2004 are held constant at 2002 levels, 2006 and 2007 are held at 2005 levels). In addition, prior to 2014, some fires were not categorized as wildfires or prescribed fires in the EPA Trends state level totals and instead the emissions were included in the Miscellaneous category.

EQUATES wild and prescribed fire emissions were based on year-specific information and used a consistent set of assumptions, tools, and input data sets (see Section 2.1.3). For example, the Bluesky Pipeline was used in EQUATES to process national fire activity into emissions inventories. A previous version of the processing tool, the Bluesky Framework, was used for many of the previous emissions platforms. There are several differences between the different versions of the Bluesky Pipeline and Bluesky Framework. A comparison of the 2017 emissions estimates from the two processing tools identified that a reduction in tons of fuel consumption for flaming, smoldering and residual smoldering fire phases in the Bluesky Pipeline compared to the Bluesky Framework had the largest impact, lowering PM_2.5_ emissions from prescribed and wildfires by 17%.

### 1.3 CO

CO emissions from all three data sources (Fig. 16) show a decreasing trend from 2002 to 2017 due to decreasing emissions from onroad vehicles (Fig. 17) and nonroad equipment. Onroad CO emissions from EQUATES are 5-16% higher than the EPA Trends data in 2002-2006 because of the switch to the more recent MOVES model. Similar to the PM_2.5_ emissions, the step changes in the EPA Trends data and the increased year-to-year variablity in the previous emissions platforms are mainly attributed to patterns in the prescribed and wildfire emissions (Fig. 18).

### 1.4 VOC

While trends in the other pollutants are driven by one or two main sources, many sources are large contributors to regulatory VOC emissions (Fig. 8), including onroad vehicles and nonroad equipment, fires, VCPs, and oil and gas sources. Differences in the estimation methods for these sources lead to the differences in the VOC trends seen in Fig. 19. Similar to NO_X_ and CO, trends in EQUATES VOC emissions from onroad vehicles are smoother and steeper than the other data sources and exibit higher emissions for 2002 to 2005 (Fig. 20). However, unlike the other pollutants, EQUATES VOC emissions are lower than the EPA Trends data by 9-28% between 2007 and 2017. The lower EQUATES emissions in more recent years is largely due to a decrease in estimated emissions from light duty vehicles in MOVES3 compared to previous MOVES versions. Similar to PM_2.5_ and CO, there are also large differences in emissions from wild and prescribed fires, with EQUATES emissions 50-60% lower than the EPA Trends or previous emissions platform data in some years (Fig. 21).

Fig. 22 shows trends in VOC emissions from VCPs. The use of the VCPy framework in EQUATES leads to differences in emissions estimates on the order of 5 to 20% for most years between 2002 and 2013. Estimates are within 5% for the final four years of the time series when the VCP data and methods are much more similar across the three data sources.

There are large differences in VOC emissions from oil and gas sources from EQUATES compared to the EPA Trends and previous modeling platforms (Fig. 23). As described earlier, oil and gas sources were missing or underestimated for many states in the 2002, 2005, and 2008 NEIs. EQUATES VOC emissions from oil and gas are approximately 50 to 300% higher than the EPA Trends data, reflecting an improved estimation of emissions from this source using the latest version of EPA's Oil and Gas Tool (see Section 2.1.10). There are also differences in the emissions estimates for the latter half of the timeseries when the Oil and Gas Tool was used in all three data sources. Over the last decade there have been multiple improvements to the tool. Different version of that tool were used for different years in the previous modeling platforms data, creating some of the year-to-year changes in the grey line. There are also year-to-year changes in the EQUATES data. This is expected for this source and reflects emissions variability due to weather conditions and changes in energy pricing, e.g., the cost of coal versus natural gas.

### 1.5 NH_3_

All three data sources show an increasing trend in NH_3_ emissions from 2014 to 2017 (Fig. 24). However, the trends are different for earlier years, with EQUATES showing a relatively flat trend, the EPA Trends data showing a decreasing trend from 2010 to 2014, and the previous modeling platform data showing an increasing trend. Differences are driven in part by differences in estimation of agricultural sources which make up over 80% of total NH_3_ emissions. Sixty to seventy percent of agricultural emissions are from livestock sources and the remaining 30–40% are from fertilizer emissions. Agricultural emissions in the EPA Trends data are included in the Miscellaneous category and so cannot be compared directly to ag emissions from EQUATES and the previous platforms. Fertilizer emissions are missing in the previous emissions platforms for 2002 through 2012 which accounts for the lower emissions in those years. (Note that these missing emissions would not have been an issue when running CMAQ with the previous emissions platforms since fertilizer emissions can be calculated online by CMAQ.) EQUATES NH_3_ emissions include fertilizer emissions calculated online in CMAQ using the birdirectional air-surface exchange of NH_3_ option in CMAQ version 5.3.2 for all years. Differences in the NH_3_ trends are also due to livestock emissions (Fig. 25). EQUATES emissions from livestock are based on 2017 values, where previous years are estimated by scaling the 2017 data using USDA animal head counts (see Section 2.1.1). The step changes in the previous emissions platforms are due to changes in the model used to estimate livestock emissions, including differences in the USDA animal population data. For example, the lower emissions in 2014 and 2015 in the platform data are due to an underestimation of the population totals for mature dairy cows (see Section 4.5.3.2 of the 2017 NEI Technical Support Document for additional details) [7]. Although agricultural emissions are the largest contributor to national total NH_3_ emissions, emissions from vehicles are an important source in urban areas and fire emissions also dictate the overall NH_3_ burden in the US. Thus, the updates to mobile source and fire emissions in EQUATES are also relevant to changes in NH_3_ trends.

The emissions comparison figures and supporting dataset show the improved consistency in the EQUATES emissions trends compared to other EPA emissions data. In particular, the improved methods used in EQUATES for estimating emissions from onroad vehicles, fires, oil and gas sources, and VCPs had a substantial impact on year-specific emissions estimates and 2002 to 2017 trends. There is a great deal of spatial variability in US emissions, and as such the differences in these aggregated data may not reflect the differences for a specific state or county. The annual and monthly total gridded EQUATES emissions files that are included in the data repository allow for additional analysis for specific regions and time periods.

## 2 Experimental Design, Materials and Methods

### 2.1 County-Level Source-Specific Emissions

Table 4 provides a summary of all the methods used to develop emissions for individual US sources. For many sources, EQUATES emissions were estimated by scaling data from the 2017 NEI with scaling factors based on activity data and/or control information. When possible, the scaling factors used to estimate emissions based on the 2017 NEI data are included in the Supplemental Material. For some emissions categories, e.g., emissions from motor vehicles, the scaling procedure is too complex to summarize here. More detailed information on the data and scaling procedures used for these source categories are included in the INV emissions files. A description of each emissions source and details on the methods used to develop the 2017 emissions are available in the NEI Technical Support Document [7]. EPA releases emissions modeling platforms for regulatory and research modeling years. An emissions modeling platform includes the National Emissions Inventory data and ancillary files to allocate the annual total emissions to the temporal and spatial scales needed by the air quality model. Additional documentation of emissions modeling methods used for EQUATES are available in the 2016 version 1 (2016v1) emissions modeling platform Technical Support Document [8]. The following sections do not repeat information available in these technical support documents, but rather explain how the 2017 NEI emissions were used to develop emissions inventories for the entire sixteen-year timeseries. For a few sources, EQUATES uses emissions modeling platform data from earlier NEIs (2002, 2005, 2008, 2011, 2014).

**Table 4:** Brief description of the method used to develop emissions for each source category.

Source Category	Point/Nonpoint/Mobile	Category Name(s)	Brief Method Description
Agriculture	nonpoint	ag	NH_3_ livestock emissions based on scaling 2017 NEI values using animal head count data.
Electrical Generating Units	point	ptegu, cem	Used existing hourly data (from multiple NEIs) for all years but processed using most recent tools/methods.
Fires	point	ptfire, ptfire_grass, ptagfire	Based on new methods (see Section 2.1.3) to produce day-specific estimates.
Fugitive Dust	nonpoint	afdust	For agricultural dust, unpaved road dust, and paved road dust, used 2017 NEI data and scaling factors based on activity surrogates. All other sources used 2017 NEI data for all years.
Mobile – Airports	mobile	airports	Used 2017 NEI data and scaling factors based on Federal Aviation Admin. Terminal Area Forecast data.
Mobile – Commercial Marine Vessels	mobile	cmv_c1c2, cmv_c3	Used 2017 NEI data and scaling factors based on regional fuel consumption as an activity surrogate with additional pollutant-specific adjustments for fuel standards.
Mobile – Nonroad	mobile	nonroad_gas, nonroad_diesel	Estimated using EPA's Motor Vehicle Emission Simulator (MOVES) version 2014b supplemented with data for California and Texas.
Mobile – Onroad	mobile	onroad_gas, onroad_diesel	Estimated using MOVES version 3 supplemented with data for California.
Mobile – Rail	mobile	rail	Used 2017 NEI data and scaling factors based on fuel sales data as an activity surrogate with additional adjustment for specific pollutants to account for regulation and sulfur technology.
Oil and Gas	point	pt_oilgas	Used year-specific modeling platform data (based on multiple NEIs).
Oil and Gas	nonpoint	np_oilgas	Used Oil and Gas Tool for 2002, 2005, 2008, 2011, 2014, 2016, 2017 and adjustment factors for all other years.
Other Nonpoint Sources -Commercial Cooking	nonpoint	nonpt	Used year-specific modeling platform data (based on multiple NEIs).
Other Nonpoint Sources -Fuel Combustion	nonpoint	nonpt	Commercial and industrial biomass used 2017 NEI data and scaling factors based on national-level consumption data. For all other emissions used year-specific modeling platform data (based on multiple NEIs).
Other Nonpoint Sources – Gas Stations	nonpoint	nonpt	Linear interpolation between 2002 NEI and 2017 NEI data.
Other Nonpoint Sources – Industrial Processes	nonpoint	nonpt	Used year-specific modeling platform data (based on multiple NEIs).
Other Nonpoint Sources - Miscellaneous	nonpoint	nonpt	Used 2017 NEI data for all years.
Other Nonpoint Sources – Waste Disposal	nonpoint	nonpt	Used 2017 NEI data for all years, except composting. For composting scaled 2017 NEI values based on activity surrogate.
Other Point Sources – Fuel Combustion	point	ptnonipm	Used year-specific modeling platform data (based on multiple NEIs).
Other Point Sources – Gas Stations	point	ptnonipm	Linear interpolation between 2002 NEI and 2017 NEI data.
Other Point Sources – Industrial Processes	point	ptnonipm	Used year-specific modeling platform data (based on multiple NEIs).
Other Point Sources – Miscellaneous	point	ptnonipm	Used 2017 NEI data for all years.
Other Point Sources – Waste Disposal	point	ptnonipm	Used 2017 NEI data for all years.
Residential Wood Combustion	nonpoint	rwc	Scaled 2017 NEI values based on national-level consumption data.
Volatile Chemical Products including Solvents	nonpoint	np_solvents	Based on new VCPy method (see section 2.1.14).

The EPA uses Source Classification Codes (SCCs) to classify different types of processes or functions that emit air pollutants, e.g., oxides of nitrogen produced by electricity generation from boilers; particulate matter from agricultural field burning; volatile organic compounds produced from the manufacture of paints. The SCCs are used as a primary identifying data element in EPA's NEIs. The SCCs used in the 2017 NEI were classified into five broad types: point, nonpoint, onroad, nonroad, and events. For EQUATES we simplify this to three broad categories that indicate how the emissions are processed and packaged: mobile (onroad + nonroad), point (point + event), and nonpoint. The point category emissions are associated with a specific location defined by longitude and latitude. Emissions for mobile and nonpoint (stationary) sources are associated with a specific area (e.g., county, country) in the inventory files. See Section 2.2 for more information on the spatial allocation of emissions to the CMAQ grid.

#### 2.1.1 Agriculture (ag)

The emissions from the agriculture (ag) source category are from livestock waste and fertilizer. The 2017 NEI was used as the reference year inventory and then scaling factors were developed to estimate emissions for 2002-2016. County-specific scaling factors for each of seven livestock categories were developed following the methods described in the 2016v1 Technical Support Document based on animal count data from the US Department of Agriculture (USDA) (Table S2). The livestock categories are cattle, including calves; cattle, cows, milk; chicken, broilers; chicken, layers; hogs; sheep, including lambs; turkeys. The 2017 NEI agriculture category includes emissions from a few animal types that are not included in this list, e.g., goats, deer. Emissions for these animal categories were held constant at 2017 levels for all years.

Scaling factors were calculated and applied retrospectively year-by-year, i.e., scaling factors were applied to 2017 to estimate 2016 emissions, then the 2016 emissions were scaled to estimate 2015, etc. Two issues with the USDA data had to be addressed. The first issue was missing data in the USDA animal count reports. Where county- or state-specific animal count data were not available, scaling factors were based on USDA national reports (Table S2). The second issue was that the method for estimating animal counts in the USDA annual reports changed over time, creating large artificial increases or decreases in the animal count data (e.g., on the order of ±50%). To avoid artificial step-changes in the scaling factors due to method changes, all scaling factors were limited to a range of 0.8 to 1.2 (±20%) from year-to-year. The choice of a 20% cut off was based on expert knowledge in the absence of more quantitative data that could constrain the scaling factors. SMOKE was used to develop month-to-hour temporal profiles for ag emissions based on the method described in Zhu et al. [9]. The same temporal profiles were applied to all livestock categories and all pollutants.

NH_3_ emissions from fertilizer in the INV packages were calculated online by CMAQ, accounting for bidirectional air-surface exchange of atmospheric NH_3_ (Table S2) and were then aggregated to a SMOKE input format and included in the INV files. An hourly temporal profile (GENTPRO) is also provided to allocate the annual emissions to hourly. However, no script for processing these files is provided since EPA did not use these emissions for input to CMAQ. Users are strongly encouraged to use the bidirectional NH_3_ option in CMAQ for estimating fertilizer NH_3_ emissions in their simulation. These files are provided for potential non-CMAQ applications.

#### 2.1.2 Electric Generating Utility (ptegu)

The annual inventories for EGUs from each of the most recent year-specific EPA emissions modeling platforms were identified for each year. Tables S3 and S4 provide the NEI year and SMOKE version used to create the previous model platform data for 2002–2017. A time-dependent list of known unit retirements in the EPA's Emissions Inventory System (Table S2) was used to ensure facilities were removed from all inventory years after their retirement. The inventories were then split into two inventories, continuous emissions monitoring (CEM) and non-CEM, based on whether the units matched the available year-specific hourly emissions data for NO_X_ and SO_2_ available from EPA's Clean Air Markets Division (Table S2). The annual hourly CEM data were processed to correct for missing or interpolated NO_X_ and SO_2_ values. Partial-year reporters were identified in the CEM data as units that submitted an incomplete year of hourly emissions data. The difference between the annual NO_X_ and SO_2_ CEM data and the annual inventory unit-specific NO_X_ and SO_2_ data were assigned to the missing months, typically winter, and temporalized to hourly values using region- and fuel-specific temporal profiles.

Region- and fuel-specific temporal profiles were calculated using the hourly CEM data from the units within each region that primarily use a specific fuel. These temporal profiles are included in the INV files. The regions were defined using multi-jurisdictional organizations boundaries and climate regions. Units identified as peaking (operating during portions of the year with the highest demand) received separate profiles compared to those that were identified as base load (largely operating the whole year). Regional fuel specific temporal profiles were then applied to the EGUs that did not have matched unit-level CEM data.

#### 2.1.3 Fires (ptagfire, ptfire, ptfire_grass)

Point source day-specific fire emissions are provided in three distinct categories: agricultural fires (ptagfire), wildfires and prescribed fires (ptfire), and grass or rangeland fires including Flint Hills, Kansas (ptfire_grass). National fire activity data were collected for 2003-2017 using the year-specific remote sensed Hazard Mapping System (HMS) fire dataset, the Geospatial Multi-Agency Coordination (GeoMAC) active fire perimeters, the Monitoring Trends in Burn Severity (MTBS) historical fire perimeters, and the Incident Status Summary (ICS-209) fire situation burn reports (Table S2). These datasets were reformatted to be ingested into the Satellite Mapping Automated Reanalysis Tool for Fire Incident Reconciliation version 2.0 (SF2) (Table S2). HMS activity data over year-specific USDA cropland data layer (CDL) agriculture and grasslands were separated before they were imported into SF2 which does not handle agriculture fires [10]. These fire detections from the HMS dataset were used to create the ptfire_grass and ptagfire inventories over the CONUS. The ICS-209 fire situation reports were pre-processed to correct end dates that occurred before start dates and set the duration of very small fires to a single day. In the 2003 data, the HMS data was augmented with Moderate Resolution Imaging Spectroradiometer (MODIS) active fire detect data for dates where HMS was not available (Table S2). Since fire activity data were not available for 2002 from the same sources as in the other years, the fire activity data were extracted from the existing 2002 NEI inventory and processed.

The activity data for the ptfire_grass source category came from the year-specific HMS activity data where the location of the satellite detect was over grasslands as identified using the CDL. Grassland-specific emissions factors were applied to the assumed fuel consumption per satellite detect to get emissions by burn location.

The Flint Hills region of Kansas is an ecologically unique region that has annual prescribed burning and is inventoried separately. In the Flint Hills region during the March through May prescribed burn season, the fire activity data were based on the year-specific prescribed burn activity in each county available from the Kansas Department of Health and the Environment (Table S2). The HMS detects were used to estimate temporal and spatial allocation of the fires with county-specific and year-specific area burned per detect used to estimate the area burned per HMS detection. Unlike the other years, the 2002 Flint Hills emissions inventory was created at the county level because HMS data were unavailable in 2002. A surrogate of National Land Cover Database grasslands was used in SMOKE processing to spatially apportion the Flint Hills fire emissions for 2002.

Bluesky Pipeline was used to process the national fire activity into emissions inventories. The Bluesky Pipeline processing used Fuel Characteristic Classification System version 1.4 spatial data to identify the fuel beds and the underlying fuel loading within those beds (Table S2). The U.S. Forest Service CONSUME software (Table S2) was then used to calculate the fuel consumption by burn phase (i.e., flaming, smoldering) for each daily fire. Finally, Fire Emission Production Simulator (FEPS) emissions factors were applied to the fuel consumption by burn phase to get emissions values by daily fire location.

#### 2.1.4 Fugitive Dust (afdust)

The anthropogenic fugitive dust source category (afdust) contains PM_10_ and PM_2.5_ emissions from paved roads, unpaved roads and airstrips, construction (residential, industrial, road and total), agriculture production, and mining and quarrying. For year 2017, the afdust category uses emissions and ancillary files from the 2017 NEI. The remaining years, 2002-2016, were based on scaling the 2017 data. Emissions from paved roads from the 2017 NEI were scaled using VMT data by county. Section 2.1.8 provides details on how the VMT data were developed. VMT was only calculated for NEI years (every three years starting with 2002), and the remaining years were interpolated. The same procedure was used to scale the 2017 emissions from unpaved roads to generate emissions for all the years. 2017 agricultural dust emissions (e.g., dust kicked up by hooves) were scaled using the same factors based on animal count data that were used to scale agriculture livestock emissions, as described Section 2.1.1. All other afdust categories, such as crop tilling and construction, were held constant throughout the time series. Emissions estimates for all years were then reduced by applying a “transport fraction,” which is based on land cover and represents the amount of afdust emissions that are trapped at the ground. See section 2.2.1 of the 2016v1 emissions modeling platform Technical Support Document on ‘Area Fugitive Dust Transport Fraction’ for further details [8]. Additionally, a meteorology adjustment was also applied to afdust emissions to account for a reduction in emissions due to precipitation. Emissions were reduced based on the amount of precipitation and snow cover within each grid cell.

#### 2.1.5 Mobile – Airports (airports)

For year 2017, airport emissions from landing, takeoff, and ground support equipment were based on the 2017 NEI. For the remaining years, scaling factors were created using the 2018 Federal Aviation Administration (FAA) Terminal Area Forecast itinerant landing and takeoff data (Table S2). Separate scaling factors were calculated for four itinerant classes: commercial aviation, general aviation, military, and air taxi. Scaling factors were calculated for each airport where a match was possible between the 2017 NEI and the Terminal Area Forecast data or at the state level when an airport-level match was not possible. When a scaling factor could be calculated at an airport by itinerant class, the change from 2017 was limited to be no greater than a 500% increase or an 80% reduction. When a scaling factor was calculated for a state by itinerant class, the change from 2017 was limited to a be no greater than a 200% or a 50% reduction. Due to the incomplete nature of the military itinerant data, state-level military class values were held flat at 2017 levels for all years.

#### 2.1.6 Mobile – Commercial Marine Vessels (cmv_c1c2, cmv_c3)

Emissions for commercial marine vessels (CMV) that use Category 1 and 2 (i.e., small to medium-sized) engines for propulsion (cmv_c1c2) and vessels that use large Category 3 engines (cmv_c3) were based off the 2016v1 modeling platform for 2016 and the 2017 NEI for 2017. The 2016v1 platform and the 2017 NEI used consistent methods for CMV [7,8]. These 2016 and 2017 datasets include CMV emissions from Environment and Climate Change Canada based on a 2015 Canada inventory. More information on these datasets is available in the 2016v1 and 2017 emissions modeling platform Technical Support Documents. The 2016 emissions were used as the reference year inventory and scaling factors were developed to estimate emissions for 2002-2015.

For cmv_c3, scaling of the 2016 emissions was done in three steps: activity adjustments, sulfur adjustments, and NO_X_ adjustments. Residual fuel oil consumption data on C3 vessels from the Energy Information Administration (EIA) of the US Department of Energy were used to calculate a scaling factor for 2002–2015 using 2016 as a reference year. Next, annual sulfur and particulate matter (PM) scaling factors were estimated to correct for changes in both national and global fuel sulfur standards. These scaling factors were created by calculating emission factors for each year based on the fuel sulfur levels using the same methods as the 2017 NEI and 2016 platform calculations. These emission rates were then ratioed against the rates for 2016. This method generated separate scaling factors for vessels operating within the US Emission Control Area (ECA) and outside it (Table S2). These scaling factors were applied to PM and SO_2_ emissions for historic years along with the activity adjustments.

Finally, NO_X_ scaling factors were derived from the proportion of main engines meeting Tier 0 through Tier 3 standards (Table S2) in each of the historic calendar years. To estimate the populations for each year, the fraction of each engine type was determined from the 2017 automatic identification system dataset used as the basis for the CMV emissions in the 2017 NEI. The fraction of the engine populations for Tiers 0, 2, and 3 were assumed to grow linearly from the first year that their respective regulations took effect, until 2017. Tier 1 vessels were assumed to make up the difference of the population such that the sum of the fractions equals 1 for all years between 2002 and 2017. The fractions of each engine type were used to estimate a fleet average NO_X_ emission rate for each year. Finally, the scaling factor for each year was determined by taking the ratio of each emission rate with the 2016 emission rate. These scaling factors were applied to NO_X_ emissions for historic years along with the activity adjustments. For non-US waters, including Canada, only the pollutant-specific factors were applied, with no change in activity applied in those areas. Table S5 provides the scaling factors for cmv_c3 emissions by region and pollutant.

Similar to the estimation of cmv_c3 emissions, scaling factors for cmv_c1c2 consist of activity-based scaling factors and pollutant-specific factors reflecting changes in cleanliness of fuels over time on a per gallon basis due to changing regulations. Activity scaling factors for cmv_c1c2 emissions were based on fuel usage data for “distillate sales/deliveries to vessel bunkering consumers” available from the EIA (Table S2). This category was chosen because C1/C2 vessels are required to use nonroad marine fuels and cannot use residual fuels as allowed for C3 vessels. State-level fuel data were found to be missing for several states or inconsistent from year-to-year. As an alternative to the state-level data, the Petroleum Administration for Defense Districts (PADD) fuel data from the EIA (Table S2) was used for developing scaling factors. Scaling factors were created by dividing the fuel data for each PADD and each year by the 2016 PADD-level fuel data. To ensure that the scaling factors could be applied to the emissions data at the county level, state and county Federal Information Processing Standard (FIPS) codes were linked to each PADD.

The cmv_c1c2 emissions were also adjusted with scaling factors to reflect changes in the vessel fleet and the implementation of fuel and emissions standards. Scaling factors were developed that accounted for regulatory Tier level changes for each year based on an estimate of fleet turn over. These values were used to weight the emission factors for each Tier standard which were compared to the 2016 aggregated emissions factors to generate scaling factors for each earlier year and each pollutant. SO_2_ and PM emissions were further adjusted to account for the introduction of ultra-low sulfur marine diesel fuel which was phased in starting in 2010. Table S6 provides the scaling factors for cmv_c1c2 emissions by region and pollutant.

#### 2.1.7 Mobile – Nonroad (nonroad)

The nonroad mobile source category (nonroad) includes all emissions sources from vehicles that operate off the road network such as construction, landscaping, and port equipment. For all states except California and Texas, nonroad emissions come from MOVES version 2014b. MOVES was run for all NEI years in the time series: 2002, 2005, 2008, 2011, 2014, 2017. The 2017 emissions are the same as 2017 NEI and the 2016 emissions are from the inventory component of the 2016v1 emissions modeling platform. For all other years, emissions were linearly interpolated between the two closest years for which MOVES outputs were available. For example, 2003 and 2004 were estimated using linear interpolation between 2002 and 2005; 2015 was estimated using linear interpolation between 2014 and 2016.

For California, the California Air Resources Board (CARB) submitted nonroad emissions for the 2016v1 platform and 2017 NEI to be used in place of the MOVES estimates. These emissions were also used for EQUATES. CARB nonroad data for 2011 and 2014 were available but had inconsistent SCCs compared to the SCCs used in 2016 and 2017 methodology. To preserve consistency in the list of SCCs used across all years, county total scaling factors were calculated using 2016 as a baseline and ratioed against the CARB 2011 and 2014 county level data. The scaling factors were applied to the CARB 2016 inventory to create CARB-based inventories for 2011 and 2014 which were consistent with 2016 and 2017. Inventories for 2012, 2013, and 2015 were estimated using linear interpolation between the 2011 and 2014 datasets. For all years prior to 2011, scaling factors were computed from the MOVES output (or MOVES-based interpolations for non-NEI years) by county, SCC, and pollutant for all of California, using 2016 as the baseline. For example, 2005 California nonroad = 2016 CARB nonroad * (2005 MOVES nonroad / 2016 MOVES nonroad) for each county-SCC-pollutant.

For Texas, the Texas Commission on Environmental Quality (TCEQ) provided nonroad emissions inventories for 2002, 2010, and 2017, in addition to their 2016 inventory from the 2016v1 platform. Inventories for those years were all used directly. Emissions for 2011-2015 were estimated using linear interpolation between 2010 and 2016, and emissions for 2003-2009 were estimated using linear interpolation between 2002 and 2010 by county and SCC.

Mobile nonroad emissions data are separated into two sectors: diesel and non-diesel. The non-diesel component includes emissions from gasoline, compressed natural gas (CNG), ethanol fuel blended with gasoline up to 85% (E-85), and PM emissions from electric vehicles from brake and tire wear. Since gasoline makes up most of the non-diesel emissions, this category is labeled “gas” in the inventory and MR files. The result is two nonroad categories: nonroad_gas and nonroad_diesel.

#### 2.1.8 Mobile – Onroad (onroad)

The onroad mobile source category (onroad) includes emissions from motorized vehicles that are normally operated on public roadways including passenger cars, motorcycles, minivans, sport-utility vehicles, light-duty trucks, heavy duty trucks, and buses. Onroad emissions are generated using a set of software tools within SMOKE (called SMOKE-MOVES). For the NEI years – 2002, 2005, 2008, 2011, 2014, 2017 – emissions are based on emission factors from MOVES version 3 (MOVES3) [11] and year-specific VMT and VPOP data.

SCC-specific emission factors (i.e., different factors based on vehicle type, fuel type, road type, and emission process) were generated by running MOVES in emissions rates mode at county scale for 328 representative counties total (290 for the Continental U.S.), two representative fuel-months (January and July) and a wide range of temperature conditions. Similar to the procedure used for the 2017 NEI, representative counties were chosen based on state, average light duty vehicle age, inspection and maintenance programs, fuel regions, and altitude.

Activity data were created for all years by using the 2017 NEI data as the reference year and creating scaling factors for earlier years. Scaling factors for VMT were based on highway statistics reports from the Federal Highway Administration (FHWA) (Table S2), which include annual vehicle miles traveled data by state and road type. Year-to-year scaling factors were calculated by state and by MOVES road type (rural restricted, rural unrestricted, urban restricted, urban unrestricted) for each year. The year-to-year approach was used to screen for step changes in VMT possibly due to a method change in how road types are classified rather than an actual change in VMT activity. For any state where the year-to-year change for any of the four road types was 10% higher or 10% lower, the road type-specific scaling factors for all four road types were replaced by a state total VMT scaling factor, as shown in the example in Table 5.

**Table 5:** Example of 2013/2014 VMT scaling factors based on FHWA data for North Carolina. In this case the road type-specific scaling factors for three of the four road types exceeded ±10% and thus scaling factors for all types were replaced by the state total VMT scaling factor of 0.97.

MOVES Road Type	2013 VMT (millions of miles)	2014 VMT (millions of miles)	2013/2014 original scaling factor	Final Scaling Factor
Rural Restricted Access	6389	7928	0.81	0.97
Rural Unrestricted Access	34221	28636	1.20	0.97
Urban Restricted Access	21409	22959	0.93	0.97
Urban Unrestricted Access	43193	48489	0.89	0.97
Total	105212	108012	0.97	

Year-to-year scaling factors were calculated for each one-year step from 2017 back to 2002, and then multiplied together to estimate VMT for the earlier years. For example: 2014 VMT = 2017 VMT * 2016/2017 scaling factor * 2015/2016 scaling factor * 2014/2015 scaling factor.

Other types of activity data, including VPOP, hoteling hours, vehicle starts, and ONI, were calculated using VMT-based ratios based on 2017, applied to the earlier year VMT. For example: 2014 VPOP = 2017 VPOP * (2014 VMT / 2017 VMT). The new 2002-2017 activity data were then summed to county-vehicle-road-month totals and split based on fuel type using fuel data from the MOVES3 model. The distribution of vehicle types for 2017 (e.g., light duty vehicles, combination long haul trucks) is based on the 2017 NEI. The distributions of vehicle types for the other years vary based on the scaling of VMT by road type, e.g., combination long haul trucks are more affected by changes in restricted road VMT than other vehicle types.

To produce emissions for the non-NEI years – 2003, 2004, 2006, 2007, 2009, 2010, 2012, 2013, 2015, 2016 – MOVES3 was run for all years in inventory mode at national default scale. Default scale uses default inputs that represent the nation (e.g., national VMT, average age distribution, average speed distribution) and is much less computationally intensive than the county-specific emissions rate mode described above. The MOVES3 emissions from these national runs were used to create national adjustment factors to scale emission factors from the closest NEI year, either one year earlier or one year later, to the non-NEI year. These national scaling factors apply separate adjustments to all fuel types, vehicle types, road types, and model species, such that the effects of year-to-year changes in fuel and vehicle distributions are also included. For example: 2009 emissions = 2008 activity * 2008 emission factors * (2009 national MOVES3 emissions / 2008 national MOVES3 emissions).

In California, annual onroad emissions inventories for all years in the time series were provided by CARB based on their latest Emission Factor Model (EMFAC2017) (Table S2). California emissions were run through SMOKE-MOVES twice for each year: once with MOVES inputs only (same as other states), and then again with adjustment applied such that the SMOKE-MOVES output emissions match the CARB inventories. The purpose of the first run was to calculate the adjustment factors that need to be applied in SMOKE-MOVES for the SMOKE-MOVES outputs to match CARB's data. CARB's data do not include emissions from NH_3_ or refueling, thus those emissions are based solely on MOVES estimates and the activity data. VOC and PM model species receive the same adjustment factors as total VOC and PM, respectively. This approach ensures that the speciation to the CARB-provided VOC and PM emissions is consistent with MOVES-based speciation.

Similar to the mobile nonroad source, onroad emissions data are separated into two sectors, diesel and gas, where “gas” includes emissions from gasoline, compressed natural gas (CNG), ethanol fuel blended with gasoline up to 85% (E-85), and PM emissions from electric vehicles from brake and tire wear. This split was also performed for California. The result is four onroad categories: onroad_gas, onroad_diesel, onroad_ca_adj_gas, and onroad_ca_adj_diesel.

#### 2.1.9 Mobile – Rail (rail)

The rail source category includes emissions from railroad locomotives powered by diesel-electric engines. Rail emissions were taken from 2017 NEI for 2017, and 2016v1 platform for 2016. 2016 emissions were used as the reference year inventory and scaling factors were developed to estimate emissions for 2002-2015. Scaling factors consist of two year-specific components that were multiplied together: an activity adjustment factor based on EIA national distillate fuel oil sales for railroad use, and pollutant-specific adjustment factors representing the amount of NO_X_, PM, VOC, and SO_2_ emissions per gallon of fuel (Table S2). For example: 2005 NO_X_ = 2016 NO_X_* (2005 activity / 2016 activity) * (2005 NO_X_ g/gal / 2016 NO_X_ g/gal). CO and NH_3_ were scaled using only the activity adjustment factors. Rail yard emissions included in the ptnonipm source category were scaled using the same factors as the rail category. Table S7 provides the activity and pollutant scaling factors for rail sources.

#### 2.1.10 Oil and Gas (np_oilgas, pt_oilgas)

Nonpoint oil and gas (np_oilgas) annual inventories were calculated using the EPA Oil and Gas Tool [7] for the following years: 2002, 2005, 2008, 2011, 2014, 2016, and 2017. The 2017 inventory is similar to the 2017 NEI, except that the 2017 NEI for np_oilgas includes state-submitted data. EQUATES np_oilgas inventory usually excludes state-submitted data to allow for consistency in the emissions estimation methods across the domain for all years of the time series. The exception is that EQUATES includes state submitted data from Pennsylvania for 2014, 2016, and 2017 because data from Pennsylvania allowed for the distinction between conventional and unconventional well emissions and generated a better emission estimate and trend for the state. The EPA Oil and Gas Tool is split into two separate modules (i.e., two separate databases): exploration activities and production activities. The exploration module refers to the emission units and processes associated with the exploration and drilling of oil and gas wells, and the production module refers to the emissions from equipment used and processes at the wellsite to then extract the product from the well and deliver it to a central collection point or processing facility.

To create inventories for years other than 2002, 2005, 2008, 2011, 2014, 2016, and 2017, interpolation factors were calculated. Inventories for the additional years were created from the closest available year. For example, 2003 emissions were estimated by scaling 2002 emissions; 2010 emissions were estimated by scaling 2011 emissions; 2015 emissions were estimated by scaling 2014 emissions.

For emissions related to oil and gas exploration, the one-year adjustment factors were based on feet drilled or well counts from RigData (Table S2). The choice of the scaling factor data depends on the nature of the Source Classification Code (SCC). For example, emissions from Drill Rigs (SCC 2310000220) were scaled based on feet drilled data, while emissions from Oil Well Completion: All Processes (SCC 2310111700) were scaled based on well counts. Separate scaling factors were applied for each state. For 2007-2015 regional data were available for Texas and Louisiana and these data were used to create regional scaling factors in place of state factors. For 2007, 2009, 2010, 2012, 2013, and 2015 separate scaling factors were used for each of the districts of the Railroad Commission (RRC) of Texas, while Louisiana was broken up by “North” and “South” regions. For example, to estimate the 2007 emissions from Drill Rigs in RRC district 10, the 2007 emissions from the Oil and Gas Tool were scaled based on the 2007 feet drilled / 2008 feet drilled in that district.

For emissions related to oil and gas production, the one-year adjustment factors were based on historical production for oil, natural gas, and coalbed methane from the EIA (Table S2). Separate state-level factors were calculated for each of the three fuel types. For example, to estimate 2015 emissions from Oil Well Heaters (SCC 2310010100) in Pennsylvania, the 2014 emissions from the Oil and Gas Tool for this SCC were scaled based on the EIA 2015 oil production / 2014 oil production in the state. For SCCs not associated with a specific fuel time, the scaling factor was based on averaging the scaling factors for oil and for gas.

Year-specific oil and gas spatial surrogates, monthly temporal profiles, and speciation profile assignments for the years 2002, 2005, 2008, 2011, 2014, 2016, and 2017 were created. These data capture, to the degree possible, the variation in spatial distribution, temporal patterns, and model species caused by the rapid evolution of this emission sector. The 2017 surrogates, monthly temporal profiles, and speciation profile assignments are the same as in the EPA 2017 platform. For the other years, ancillary files from the closest year were used, e.g., 2002 spatial surrogates, monthly temporal profiles, and speciation profile assignments were used for 2003. In some cases, the existing monthly temporal profiles did not cover all sources in the state-submitted emissions from Pennsylvania. In these cases, monthly profiles were based on EIA historical data (see Table S2 column for “np_oilgas” sector for EIA production data available at monthly time resolution).

For the 2010 through 2017 modeling years, the North American Industry Classification System (NAICS) codes were used to separate point oil and gas emissions from other point sources in the NEI and were included in their own source category, pt_oilgas. For 2010–2017, pt_oilgas emissions inventories from the emissions platforms listed in Table S4 were used as-is. Existing emissions platforms for the years 2002–2009 did not have a separate pt_oilgas category; instead, those emissions were included in the ptnonipm category. The NAICS codes that were used to create the 2010–2017 pt_oilgas inventories in those existing platforms were applied to the 2002–2009 ptnonipm inventories to create a separate pt_oilgas source category for all years for this project. The NAICS codes in the 2002 and 2003 ptnonipm inventories are incomplete, resulting in some oil and gas emissions remaining in the EQUATES ptnonipm category rather than being moved to pt_oilgas. While this inconsistency impacts source category-based emissions summaries, the total emissions from ptnonipm and pt_oilgas are unchanged since emissions in these two categories are processed the same in SMOKE.

#### 2.1.11 Other Nonpoint Sources (nonpt)

The “other” nonpoint source category (nonpt) includes nonpoint emissions sources not split out into a separate category. This includes emissions from fuel combustion (e.g., coal, oil, and natural gas industrial boilers), industrial processes (e.g., mining, cement manufacturing), commercial cooking, waste disposal, and gas stations. The EQUATES nonpt category is consistent with the 2017 NEI nonpt category with the exception that emissions from VCPs, including solvents and asphalt paving, which are in their own source category in the EQUATES emissions called np_solvents. This source category is explained further in Section 2.1.14.

For 2002–2016, emissions for nonpt sources are primarily taken from older emissions modeling platforms (Table S4). The nonpt source category includes state submitted data that are developed using different emissions estimation methods across states and across years. The emissions from these previous emissions modeling platforms were reviewed to identify data with large year-to-year variability due to differences in the emissions modeling processes used, rather than real-world changes in the sources. To limit these types of artificial step changes, the following nonpt sources were kept flat at 2017 levels throughout the time series: all waste disposal except composting, miscellaneous non-industrial, bulk gasoline terminals, and any construction dust or agriculture dust and waste which is not part of the afdust or ag categories. In addition, emissions from the following nonpt sources were estimated using scaling factors or linear interpolation: biomass fuel combustion from industrial and commercial sources, composting, gas station refueling. The estimation methods for these three sources are described in the following paragraph. All other nonpt emissions were used as-is from the older emissions platforms. This includes all fuel combustion except biomass, all industrial processes, and commercial cooking.

Emissions for biomass fuel combustion used the 2017 NEI as the reference year. Activity scaling factors for 2002-2016 were developed for biomass fuel combustion based on State Energy Data System (SEDS) data from the EIA for commercial wood consumption (WDCCB) and industrial wood consumption (WDICB) for states in the CONUS (Table S2). State level consumption data were summed to create national scaling factors which were applied to all pollutants to represent broad trends in biomass fuel combustion driven by meteorological and economic factors. Scaling factors for composting emissions for 2002-2016 were based on linear interpolation of activity data for 2005, 2010-2014 (reported in kt of waste composted per year) provided in Table 7–19 of The EPA report “Inventory of U.S. Greenhouse Gas Emissions and Sinks: 1990-2014” (Table S2). The scaling factors for biomass fuel combustion and waste composting are provided in Table S8. Nonpt refueling emissions from gas stations were taken from existing modeling platform data for 2002 (Table S4) and the 2017 NEI and then 2003-2016 emissions were estimated using linear interpolation. Separate interpolation scaling factors are applied for each individual SCC associated with gas stations.

#### 2.1.12 Other Point Sources (ptnonipm)

The other point source category includes point emissions sources not split out into a separate category, e.g., fuel combustion, industrial processes, commercial cooking, waste disposal, and gas stations. Like nonpt, the EQUATES ptnonipm category is consistent with the 2017 NEI ptnonipm category with the exception that emissions from VCPs have been moved to np_solvents.

For 2002-2016, most ptnonipm sources are taken from existing inventories (Table S4), including all fuel combustion and all industrial processes. Mirroring the nonpt methodology, the following ptnonipm sources were kept flat at 2017 levels throughout the time series: all waste disposal, miscellaneous non-industrial, bulk gasoline terminals, and any point source construction dust or agriculture dust and waste which is not part of the afdust or ag categories. Point refueling emissions from gas stations were taken from existing datasets for 2002 and 2017, and then linearly interpolated for 2003-2016.

Rail yard emissions were taken from 2017 NEI for 2017, and 2016 version 1 platform for 2016. The 2016 emissions were scaled to 2002-2015 using the same factors that were used to scale emissions from the rail source category (Section 2.1.9).

#### 2.1.13 Residential Wood Combustion (rwc)

The source category rwc includes residential wood burning devices such as fireplaces, fireplaces with inserts, free standing woodstoves, pellet stoves, outdoor hydronic heaters, indoor furnaces, and outdoor burning in firepits and chimineas. The 2017 NEI rwc emissions were used as-is for 2017. Methods for estimating rwc have changed substantially between NEI years and this includes multiple updates in methodology for rwc in the 2017 NEI, including updated activity to better account for outdoor recreational burning and improved characterization of central heaters from both cordwood and pellet-fired hydronic heaters and furnaces. To avoid large changes in emissions estimates due to changing methodology, the 2017 emissions were used as a reference year and national scaling factors were developed for 2002-2016.

Scaling factors were based on SEDS data from EIA for wood energy consumed by the residential sector (WDRCB) (Table S2). State level consumption data were found to be very noisy, with interannual fluctuations spanning -90% to more than +1000%. The state level data were thus summed for states in the CONUS to create national scaling factors which were applied to all pollutants to represent broad trends in rwc driven by meteorological and economic factors. Regulatory actions impacting rwc emissions (e.g., EPA's 2015 Residential Wood Heater New Source Performance Standards) are reflected in the 2017 emissions but are not directly incorporated in the scaling methodology due to a lack of consistent data across all states and years. Scaling factors for rwc are summarized in Table S9.

Modeled minimum daily temperature was used to develop year-to-day temporal profiles for portions of the rwc source category following the methodology described in the Technical Support Document for the 2016v1 emissions modeling platform [8].

#### 2.1.14 Volatile Chemical Products Including Solvents (np_solvents)

The nonpoint solvent source category (np_solvents) is a diverse collection of sources whose emissions are driven by evaporation. Included in this category are everyday items, such as cleaners, personal care products, adhesives, architectural and aerosol coatings, printing inks, and pesticides. These sources exclusively emit organic gases and span residential, commercial, institutional, and industrial settings. The organic gases that evaporate from these sources often function as solvents in products but may serve other functions (e.g., propellants, fragrances, emollients) as well. The term volatile chemical products is used synonymously with solvents to refer to this source category.

Emissions from np_solvents were derived using the VCPy framework described in Seltzer et al. [2]. The VCPy framework is based on the principle that the magnitude and speciation of organic emissions from this sector are directly related to (1) the mass of chemical products used, (2) the composition of these products, (3) the physicochemical properties of their constituents that govern volatilization, and (4) the timescale available for these constituents to evaporate. National product usage was preferentially estimated using economic statistics from the U.S. Census Bureau's Annual Survey of Manufacturers, commodity prices from the U.S. Department of Transportation's 2012 Commodity Flow Survey and the U.S. Census Bureau's Paint and Allied Products Survey, and producer price indices, which scale commodity prices to target years and are retrieved from the Federal Reserve Bank of St. Louis. In circumstances where the aforementioned datasets were unavailable, default usage estimates were derived using functional solvent usage reported by a business research company or in sales reported in a CARB California-specific survey. The composition of products was estimated by generating composites from various CARB surveys and profiles reported in the U.S. EPA's SPECIATE database [4,5]. For oil and gas solvent usage, the composition is assumed to be dominated by methanol and other hydrocarbon blends. The physicochemical properties of all organic components were generated from the quantitative structure-activity relationship model OPERA [12] and the characteristic evaporation timescale of each component was estimated using previously published methods [13,14].

National-level emissions were then allocated to the county-level using several proxies. Most emissions were allocated using population as a spatial surrogate. This includes all cleaners, personal care products, adhesives, architectural coatings, and aerosol coatings. Industrial coatings, allied paint products, printing inks, and dry-cleaning emissions are allocated using county-level employment statistics from the U.S. Census Bureau's County Business Patterns and follow the same mapping scheme used in the 2017 NEI [7]. Agricultural pesticides are allocated using county-level agricultural pesticide use as taken from the 2017 NEI, and oil and gas emissions are allocated using oil and gas well counts. References for the data sources described in this section are included in Seltzer et al. [2].

#### 2.1.15 Canada and Mexico Emissions

Canada and Mexico emissions are only included in the MR summary files. Fire emissions for Canada and Mexico are based on the Fire Inventory from the National Center for Atmospheric Research (FINN) version 1.5 (Table S2). The SMOKE system is used to process The FINN emissions for the 12US1 domain. All other Canada emissions were developed by Environment and Climate Change Canada (ECCC) as part of their Air Pollution Emission Inventory (APEI) (Table S2). SMOKE-ready emissions were developed by ECCC following Sassi et al. [15] and SMOKE processing was performed by EPA to generate MR emissions files.

Onroad Mexico emissions for 2002, 2011, 2014, and 2017 are based on monthly inventories generated by a version of the MOVES model for Mexico, which are then processed through SMOKE. Onroad emissions for Mexico for all other years are based on linear interpolation. All point and nonpoint Mexico emissions are based on a 2016 emissions inventory provided by Mexico's Secretariat of Environment and Natural Resources (SEMARNAT) and included in EPA's 2016v2 emissions modeling platform [16]. The 2016 emissions are used for 2014, 2015, and 2017. Scaling factors were created for 2002-2013 based on the long-term global emissions record from the Community Emissions Data System (CEDS) [17]. The scaling factors are created for seven broad sector categories: ships, agriculture, residential, industry, energy, transport, other. At the time the EQUATES emissions were processed, the CEDS record ended in 2014. As such, the scaling factors were calculated using 2014 as a reference year. For example, 2013 ag emissions = (2016 ag emissions from SEMARNAT) * (2013/2014 ag scaling factor based on CEDS totals for Mexico).

### 2.2 County-to-Grid Spatial Allocation

Spatial allocation of nonpoint sources to the 12US1 domain is based on using spatial surrogates, e.g., population, to distribute county-level emissions to the 12 km × 12 km CMAQ grid. Spatial allocation of EQUATES emissions was performed with multiple sets of spatial surrogates. The representative years for the spatial surrogates for each source category are provided in Table S10. A limitation of this dataset is that the spatial surrogates (e.g., population, total road miles, housing), were not year-specific but based on a limited number of years for the entire 2002-2017 time period. In general, the spatial surrogates used include:

• 2014-based US surrogates, generally used with pre-2017 emission inventories
• 2017-based US surrogates, generally used with 2017 emission inventories, including those scaled from 2017
• Year-specific US surrogates for oil and gas

For the US, one important consideration is the state and county FIPS codes. County names and FIPS codes changed for one county in South Dakota between 2014 and 2017 (formerly Shannon County / 46113, now Oglala Lakota County / 46102). Some inventories used in EQUATES have the old state/county FIPS codes, and some have the new state/county FIPS codes. Similarly, the 2014-based surrogates have the old state/county FIPS codes, and the 2017-based surrogates have the new state/county FIPS codes. For sources that have emissions in this county, it is important to use spatial surrogates that have the same state/county FIPS codes as the inventory, or else emissions from that county would be dropped by SMOKE. In the nonpt source category, since the inventories partially came from the 2017 NEI and partially from older NEIs, all state/county FIPS codes in all nonpt inventories were changed to reflect the 2017 state/county FIPS codes. The INV emissions packages include additional information on the specific spatial surrogates used for each source category, including sample scripts for performing the spatial allocation processing with SMOKE for each year. The ancillary folder of the INV packages includes the cross-reference between surrogate codes and SCC codes. Descriptions of each surrogate code are provided in tables in section 3.4 of the 2016v1 Emissions Modeling Platform Technical Support Document.

## Ethics Statements

This research meets the ethical requirements for publication in Data in Brief. This work does not involve studies with animals and humans, or data collected from social media platforms.

## Funding

The EPA through its Office of Research and Development (ORD) funded and conducted this research. ERG work was done under EPA contracts 68HERD19A0001 and EP-C-17-011. GDIT work was done under EPA contracts HHSN316201200013W and GS00F057CA. CMAS work was done under EPA contract EP-W-16-014.

## CRediT authorship contribution statement

**Kristen M. Foley:** Project administration, Methodology, Validation, Formal analysis, Data curation, Writing – original draft, Visualization. **George A. Pouliot:** Project administration, Methodology, Validation, Formal analysis, Data curation, Writing – original draft, Visualization. **Alison Eyth:** Project administration, Methodology, Validation, Funding acquisition, Writing – review & editing. **Michael F. Aldridge:** Methodology, Formal analysis, Validation, Data curation, Writing – original draft, Writing – review & editing. **Christine Allen:** Methodology, Formal analysis, Validation, Data curation, Writing – original draft. **K. Wyat Appel:** Methodology, Formal analysis, Validation, Writing – review & editing. **Jesse O. Bash:** Methodology, Formal analysis, Validation, Writing – review & editing. **Megan Beardsley:** Methodology, Formal analysis, Validation, Writing – review & editing. **James Beidler:** Methodology, Formal analysis, Validation, Data curation, Writing – original draft. **David Choi:** Methodology, Formal analysis, Validation, Writing – review & editing. **Caroline Farkas:** Methodology, Formal analysis, Validation, Writing – review & editing. **Robert C. Gilliam:** Methodology, Formal analysis, Validation, Writing – review & editing. **Janice Godfrey:** Methodology, Formal analysis, Validation, Writing – review & editing. **Barron H. Henderson:** Methodology, Formal analysis, Validation, Writing – review & editing. **Christian Hogrefe:** Methodology, Formal analysis, Validation, Writing – review & editing. **Shannon N. Koplitz:** Methodology, Formal analysis, Validation, Writing – review & editing. **Rich Mason:** Methodology, Formal analysis, Validation, Writing – review & editing. **Rohit Mathur:** Methodology, Formal analysis, Validation, Writing – review & editing. **Chris Misenis:** Methodology, Formal analysis, Validation, Writing – review & editing. **Norm Possiel:** Project administration, Methodology, Validation, Funding acquisition, Writing – review & editing. **Havala O.T. Pye:** Methodology, Formal analysis, Validation, Writing – review & editing. **Lara Reynolds:** Methodology, Formal analysis, Validation, Data curation, Writing – original draft. **Matthew Roark:** Methodology, Formal analysis, Validation, Data curation, Writing – original draft. **Sarah Roberts:** Methodology, Formal analysis, Validation, Writing – review & editing. **Donna B. Schwede:** Conceptualization, Supervision, Funding acquisition, Writing – review & editing. **Karl M. Seltzer:** Methodology, Formal analysis, Validation, Data curation, Writing – original draft. **Darrell Sonntag:** Methodology, Formal analysis, Validation, Writing – review & editing. **Kevin Talgo:** Methodology, Formal analysis, Validation, Data curation, Writing – original draft. **Claudia Toro:** Methodology, Formal analysis, Validation, Writing – review & editing. **Jeff Vukovich:** Methodology, Formal analysis, Validation, Writing – review & editing. **Jia Xing:** . **Elizabeth Adams:** Data curation.

## Declaration of Competing Interest

The authors declare that they have no known competing financial interests or personal relationships which have or could be perceived to have influenced the work reported in this article.

## Data Availability

US EPA, 2022, EQUATESv1.0: Inventory Emissions (SMOKE inputs) -- 2002-2017 US_12km (Original data) (Dataverse).

## Appendix Supplementary materials

Image, application 1
