# Supplementary material for: 2002–2017 anthropogenic emissions data for air quality modeling over the United States
Source: Data Brief. 2023 Mar 2;47:109022. doi: 10.1016/j.dib.2023.109022 (PMC10023994; doi:10.1016/j.dib.2023.109022)
Supplement: Supplementary file 1 [file mmc1.docx]

**SUPPLEMENTAL MATERIAL for Data in Brief Article 2002-2017 Anthropogenic Emissions Data for Air Quality Modeling over the United States**

Supplemental Data is available on the CMAS Data Warehouse Google Drive.

Direct URL to supplemental data: <https://drive.google.com/drive/folders/1G2_LBLy7_n91Ur0ulsLZ9zwGs3luTzn2>

**Table S1.** Description of how inventory (INV) emissions data files are organized. {YYYY} indicated 4-digit year; {YY} indicates last 2-digits of year; {MM} indicates 2-digit month; {DD} indicates 2-digit day; {source} indicates emissions sector category name; * indicates wildcard character. INV emissions tar packages are available from the CMAS Center Data Warehouse DataVerse repository: <https://doi.org/10.15139/S3/MW9OLB>

| **Folder/File Name** | **File Format** | **Description** |
| --- | --- | --- |
| Tar Package Name (Size): EQUATES_INV_{YYYY}_version1.0.tar.gz (6.7 GB per year) | | |
| scripts/* | ASCII files | Sample SMOKE scripts |
| inputs/{source}/* | FF10 formatted .csv files | Annual total emissions data organized by source into 22 subfolders using the SMOKE Flat File 2010 (FF10) format |
| Tar Package Name (Size): met{YYYY}.tar.gz (43 GB per year) | | |
| METCRO2D_{YY}{MM}{DD} | Compressed netCDF | Hourly, gridded, meteorology data needed for SMOKE processing |
| Tar Package Name (Size): ancillary_data_version1.0.tar.gz (5.3 GB) | | |
| ge_dat/* | ASCII | SMOKE ancillary inputs including cross reference files, temporal profiles, speciation profiles, inventory table files |
| ocean_cl2/* | netCDF | Gridded ocean chlorine file for 3 US domains |
| smoke4.8_helper_scripts/* | ASCII | Sample common scripts used by the year specific processing scripts |
| spatial_surrogates/* | ASCII | Spatial surrogate files used by SMOKE |

**Table S2.** Data sources for source specific emissions described in Section 2.1 and 3.1.

|  | **Source Category** | **Data Source** | **Website** |
| --- | --- | --- | --- |
| 1 | ag | US Department of Agriculture National Agriculture Statistics Service Quick Stats | <https://quickstats.nass.usda.gov/> |
| 2 | ag | U.S. Department of Agriculture National Agriculture Projection Reports | [https://usda.library.cornell.edu/concern/publications/qn59q396v?locale=en#release-items 2021](https://usda.library.cornell.edu/concern/publications/qn59q396v?locale=en#release-items) |
| 3 | ag | Bidirectional air-surface exchange of atmospheric NH_3_ in CMAQv5.3 series (including CMAQv5.3.2) | <https://github.com/USEPA/CMAQ/blob/5.3.2/DOCS/Release_Notes/stage_overview.md> |
| 4 | ptegu | U.S. Environmental Protection Agency Emissions Inventory System | <https://www.epa.gov/air-emissions-inventories/emissions-inventory-system-eis-gateway> |
| 5 | ptegu | CAMD website for hourly NOx and SO2 data | https://ampd.epa.gov/ampd/ |
| 6 | ptagfire, ptfire, ptfire_grass | National Oceanic and Atmospheric Administration Hazard Mapping System | <https://www.ospo.noaa.gov/Products/land/hms.html> |
| 7 | ptagfire, ptfire, ptfire_grass | Geospatial Multi-Agency Coordination wildfire perimeter data | [https://data-nifc.opendata.arcgis.com/datasets/historic-perimeters-combined-2000-2018 2021](https://data-nifc.opendata.arcgis.com/datasets/historic-perimeters-combined-2000-2018) |
| 8 | ptagfire, ptfire, ptfire_grass | Monitoring Trends in Burn Severity (MTBS) historical fire perimeters | [https://www.mtbs.gov/direct-download 2021](https://www.mtbs.gov/direct-download) |
| 9 | ptagfire, ptfire, ptfire_grass | Website for ICS 9 fire situation burn reports | <https://famit.nwcg.gov/applications/SIT209> |
| 10 | ptagfire, ptfire, ptfire_grass | Satellite Mapping Automated Reanalysis Tool for Fire Incident Reconciliation version 2.0 | <https://firesmoke.ca/smartfire> |
| 11 | ptagfire, ptfire, ptfire_grass | National Aeronautics and Space Administration Moderate Resolution Imaging Spectroradiometer (MODIS) active fire detect data available from the Fire Information for Resource Management System | <https://firms.modaps.eosdis.nasa.gov/active_fire/> |
| 12 | ptagfire, ptfire, ptfire_grass | Website for burn activity for Flint Hills from KS Dept of Health and the Envir. | <https://ksfire.org> |
| 13 | ptagfire, ptfire, ptfire_grass | Fuel Characteristic Classification System version 1.4 spatial data | <https://landfire.gov/fccs.php> |
| 14 | ptagfire, ptfire, ptfire_grass | U.S. Forest Service CONSUME software | <https://www.fs.usda.gov/pnw/projects/consume-fuel-consumption-and-emissions-software> |
| 15 | airports | Federal Aviation Administration Terminal Area Forecast Data | <https://www.faa.gov/data_research/aviation/taf/> |
| 16 | cmv_c3 | U.S. Environmental Protection Agency Designation of the North American Emission Control Area for Marine Vessels | <https://www.epa.gov/regulations-emissions-vehicles-and-engines/designation-north-american-emission-control-area-marine> |
| 17 | cmv_c3 | U.S. Environmental Protection Marine Compression-Ignition Engines Exhaust Emissions Standards EPA-420-B-20-021 | https://www.epa.gov/regulations-emissions-vehicles-and-engines/final-rule-control-emissions-new-marine-compression-0 |
| 18 | cmv_c1c2 | U.S. Energy Information Administration fuel usage data for distillate sales/deliveries to vessel bunkering consumers | https://www.eia.gov/dnav/pet/hist/LeafHandler.ashx?n=PET&s=KD0VVBNUS1&f=A |
| 19 | cmv_c1c2 | U.S. Energy Information Administration fuel usage data for distillate sales/deliveries to vessel bunkering consumers by Petroleum Administration for Defense Districts (PADD) | https://www.eia.gov/petroleum/supply/monthly/ |
| 20 | onroad | U.S Department of Transportation Federal Highway Administration Highway Statistics Series Publications for 2002-2017, Vehicle-miles of travel, by functional system State Tables (VM-2 Tables) | <https://www.fhwa.dot.gov/policyinformation/statistics.cfm> |
| 21 | onroad | California Air Resources Board Emission Factor Model (EMFAC) 2017 version | <https://ww2.arb.ca.gov/our-work/programs/mobile-source-emissions-inventory/msei-modeling-tools> |
| 22 | rail | U.S. Energy Information Administration Distillate Fuel Oil and Kerosene Sales for Railroad Use | <https://www.eia.gov/dnav/pet/PET_CONS_821USE_A_EPD0_VRR_MGAL_A.htm> |
| 23 | rail | U.S. Environmental Protection Agency Locomotive Exhaust Emissions Standards. EPA-420-B-16-024 | <https://www.epa.gov/emission-standards-reference-guide/epa-emission-standards-nonroad-engines-and-vehicles> |
| 24 | rail | U.S. Environmental Protection Agency 2016 version 1 Emissions Modeling Platform Technical Support Document | <https://www.epa.gov/air-emissions-modeling/2016-version-1-technical-support-document> |
| 25 | np_oilgas | RigData for feet drilled and well counts | https://www.rigdata.com |
| 26 | np_oilgas | U.S. Energy Information Administration production data for oil, natural gas and CBM from EIA for scaling factors | Historical Natural Gas (http://www.eia.gov/dnav/ng/ng_sum_lsum_a_epg0_fgw_mmcf_a.htm)  Historical Crude Oil (http://www.eia.gov/dnav/pet/pet_crd_crpdn_adc_mbbl_a.htm)  Historical CBM (https://www.eia.gov/dnav/ng/ng_prod_coalbed_s1_a.htm) |
| 27 | nonpt | U.S. Energy Information Administration State Energy Data System Data for Commercial Wood Consumption in Btu (WDCCB) for 2002-2017 | <https://www.eia.gov/state/seds/seds-data-complete.php?sid=US#Consumption> |
| 28 | nonpt | U.S. Waste Composted, Table 7-19 of EPA 430-R-16-002 | https://www.epa.gov/sites/production/files/2017-04/documents/us-ghg-inventory-2016-main-text.pdf |
| 29 | rwc | U.S. Energy Information Administration State Energy Data System Data for Residential Wood Consumption in Btu (WDRCB) for 2002-2017 | <https://www.eia.gov/state/seds/seds-data-complete.php?sid=US#Consumption> |
| 30 | CA and MX fires | Fire INventory from the National Center for Atmospheric Research (FINN) | https://www2.acom.ucar.edu/modeling/finn-fire-inventory-ncar |
| 31 | CA anthropogenic emissions | Environment and Climate Change Canada Air Pollutant Emissions Inventory (APEI) | <https://www.canada.ca/en/environment-climate-change/services/pollutants/air-emissions-inventory-overview.html> |
| 32 | All sources | EPA’s Air Pollution Emissions Trends data  February 10, 2022 version of state_tier1_caps.xlsx | <https://www.epa.gov/air-emissions-inventories/air-pollutant-emissions-trends-data> |

**Table S3.** NEI year and SMOKE version used to create the emissions modeling platform data used for the ptegu sources and some ptnonipm sources in EQUATES and used as the previous model platform data used in the emissions comparison data file and figures 11 – 25 in the main text.

| **Year** | **NEI Version** | **Date emissions processed** | **Emissions Modeling Platform Technical Support Document (TSD)** |
| --- | --- | --- | --- |
| 2002 | 2002 NEI v3 | April 2007 |  |
| 2003 | 2002 NEI v3 | April 2007 |  |
| 2004 | 2005 NEI v2 | December 2010 |  |
| 2005 | 2005 NEI v2 | December 2010 | 2005 TSD: https://www.epa.gov/air-emissions-modeling/2005-version-43-platform |
| 2006 | 2005 NEI v2 | December 2010 |  |
| 2007 | 2008 NEI v2 | July 2012 | 2007 TSD: https://www.epa.gov/air-emissions-modeling/20072008-version-5-air-emissions-modeling-platforms |
| 2008 | 2008 NEI v2 | July 2012 |  |
| 2009 | 2008 NEI v2 | January 2013 |  |
| 2010 | 2008 NEI v2 | January 2013 |  |
| 2011 | 2011 NEI v2 | July 2017 | 2011 TSD: https://www.epa.gov/air-emissions-modeling/2011-version-63-platform |
| 2012 | 2011 NEI v2 | March 2015 |  |
| 2013 | 2011 NEI v2 | July 2016 |  |
| 2014 | 2014 NEI v2 | November 2017 | 2014 TSD: https://www.epa.gov/air-emissions-modeling/2014-version-71-platform |
| 2015 | 2014 NEI v2 | January 2018 | 2015 TSD: https://www.epa.gov/air-emissions-modeling/2015v71-alpha-platform |
| 2016 | 2014 NEI v2 | June 2018 | 2016v1 TSD: https://www.epa.gov/air-emissions-modeling/2016v1-platform |
| 2017 | 2017 NEI v1 | April 2020 | 2017 NEI TSD: https://www.epa.gov/sites/default/files/2021-02/documents/nei2017_tsd_full_jan2021.pdf |

**Table S4**. NEI year and SMOKE version used to create the emissions modeling platform data used for some nonpt sources in EQUATES and used in the previous modeling platform data used in the emissions comparison data file and figures 11 – 25 in the main text.

| **Year** | **NEI Version** | **Date emissions processed** | **Emissions Modeling Platform Technical Support Document (TSD)** |
| --- | --- | --- | --- |
| 2002 | 2005 NEI v2 | May 2011 |  |
| 2003 | 2005 NEI v2 | May 2011 |  |
| 2004 | 2005 NEI v2 | May 2011 |  |
| 2005 | 2005 NEI v2 | May 2011 | 2005 TSD: https://www.epa.gov/air-emissions-modeling/2005-version-43-platform |
| 2006 | 2008 NEI v2 | July 2012 |  |
| 2007 | 2008 NEI v2 | July 2012 | 2007 TSD: https://www.epa.gov/air-emissions-modeling/20072008-version-5-air-emissions-modeling-platforms |
| 2008 | 2008 NEI v2 | July 2012 |  |
| 2009 | 2008 NEI v2 | July 2012 |  |
| 2010 | 2011 NEI v2 | August 2017 |  |
| 2011 | 2011 NEI v2 | August 2017 | 2011 TSD: https://www.epa.gov/air-emissions-modeling/2011-version-63-platform |
| 2012 | 2011 NEI v2 | January 2016 |  |
| 2013 | 2011 NEI v2 | January 2016 |  |
| 2014 | 2014 NEI v2 | January 2018 | 2014 TSD: https://www.epa.gov/air-emissions-modeling/2014-version-71-platform |
| 2015 | 2014 NEI v2 | January 2018 | 2015 TSD: https://www.epa.gov/air-emissions-modeling/2015v71-alpha-platform |
| 2016 | 2014 NEI v2 | September 2019 | 2016v1 TSD: https://www.epa.gov/air-emissions-modeling/2016v1-platform |
| 2017 | 2017 NEI v1 | April 2020 | 2017 NEI TSD: https://www.epa.gov/sites/default/files/2021-02/documents/nei2017_tsd_full_jan2021.pdf |

**Table S5.** Scaling factors for cmv_c3 by region and pollutant.

| **Region** | **US East Coast** | **US South Pacific** | **US North Pacific** | **US Gulf** | **US Great Lakes** | **Global** | **ECA** | **Global** | **ECA** | **US** |
| --- | --- | --- | --- | --- | --- | --- | --- | --- | --- | --- |
| **Factors** | **Activity** | **Activity** | **Activity** | **Activity** | **Activity** | **Sulfur** | **Sulfur** | **PM** | **PM** | **NOX** |
| 2000 | 0.813937 | 1.120353 | 1.034527 | 1.006137 | 1.762816 | 1.285714 | 15 | 1.185933 | 1.707683 | 1.072302 |
| 2001 | 0.850875 | 1.151076 | 0.986164 | 0.945929 | 1.633803 | 1.285714 | 15 | 1.185933 | 1.707683 | 1.069324 |
| 2002 | 0.877217 | 1.18392 | 0.967535 | 0.903852 | 1.476676 | 1.285714 | 15 | 1.185933 | 1.707683 | 1.066345 |
| 2003 | 0.945496 | 1.157311 | 1.074991 | 0.933756 | 1.714427 | 1.285714 | 15 | 1.185933 | 1.707683 | 1.063367 |
| 2004 | 0.969609 | 1.173064 | 1.088917 | 1.036051 | 1.738377 | 1.285714 | 15 | 1.185933 | 1.707683 | 1.060388 |
| 2005 | 1.008493 | 1.173016 | 1.117765 | 1.003814 | 1.65228 | 1.285714 | 15 | 1.185933 | 1.707683 | 1.057409 |
| 2006 | 1.00721 | 1.265957 | 1.078467 | 1.021138 | 1.626075 | 1.285714 | 15 | 1.185933 | 1.707683 | 1.054431 |
| 2007 | 0.954794 | 1.182464 | 1.095163 | 0.994735 | 1.544988 | 1.285714 | 15 | 1.185933 | 1.707683 | 1.051452 |
| 2008 | 0.879394 | 1.011501 | 0.96237 | 0.913142 | 1.265169 | 1.285714 | 15 | 1.185933 | 1.707683 | 1.048474 |
| 2009 | 0.819251 | 0.969566 | 0.907615 | 0.898267 | 1.007064 | 1.285714 | 15 | 1.185933 | 1.707683 | 1.045495 |
| 2010 | 0.874853 | 1.017564 | 1.011967 | 0.954195 | 1.138309 | 1.285714 | 10 | 1.185933 | 1.454939 | 1.042517 |
| 2011 | 0.845083 | 0.940838 | 0.953861 | 0.901893 | 0.945224 | 1.285714 | 10 | 1.185933 | 1.454939 | 1.03571 |
| 2012 | 0.870967 | 0.945744 | 0.906007 | 0.897638 | 0.911052 | 1 | 10 | 1 | 1.454939 | 1.028903 |
| 2013 | 0.898515 | 0.95504 | 0.923055 | 0.918259 | 0.93323 | 1 | 10 | 1 | 1.454939 | 1.022096 |
| 2014 | 0.928719 | 0.965815 | 0.947545 | 0.944839 | 0.956794 | 1 | 10 | 1 | 1.454939 | 1.015289 |
| 2015 | 0.965052 | 0.982969 | 0.974737 | 0.97185 | 0.980011 | 1 | 1 | 1 | 1 | 1.008482 |
| 2016 | 1 | 1 | 1 | 1 | 1 | 1 | 1 | 1 | 1 | 1 |

**Table S6.** Scaling factors for cmv_c1c2 by region and pollutant.

| **2016** | 1 | 1 | 1 | 1 | 1 | 1 | 1 | 1 | 1 | 1 | 1 | 1 | 1 | 1 | 1 | 1 | 1 | 1 | 1 | 1 | 1 |
| --- | --- | --- | --- | --- | --- | --- | --- | --- | --- | --- | --- | --- | --- | --- | --- | --- | --- | --- | --- | --- | --- |
| **2015** | 1.120824 | 1.110779 | 0.927806 | 1.010391 | 1.129412 | 0.474359 | 1.09765 | 1 | 1 | 1.019802 | 1.019802 | 1.019495 | 1.019495 | 1.015383 | 1.015383 | 1.012 | 1.012 | 1 | 1 | 1.015097 | 1.015097 |
| **2014** | 0.831218 | 0.696382 | 0.581934 | 0.775984 | 0.724997 | 0.512821 | 0.70377 | 1 | 1 | 1.039604 | 1.039604 | 1.038991 | 1.038991 | 1.030766 | 1.030766 | 1.024 | 1.024 | 1 | 1 | 1.030193 | 1.030193 |
| **2013** | 0.731525 | 0.589843 | 0.589355 | 0.982874 | 0.701346 | 0.474359 | 0.798378 | 1 | 1 | 1.059406 | 1.059406 | 1.058486 | 1.058486 | 1.046149 | 1.046149 | 1.036 | 1.036 | 1 | 1 | 1.04529 | 1.04529 |
| **2012** | 1.147769 | 0.552584 | 0.589383 | 1.015118 | 0.723406 | 0.217949 | 0.901366 | 1 | 1 | 1.071507 | 1.071507 | 1.071101 | 1.071101 | 1.059056 | 1.059056 | 1.048 | 1.048 | 1 | 1 | 1.04529 | 1.04529 |
| **2011** | 0.956617 | 0.569664 | 0.651289 | 1.115821 | 1.096209 | 0.025641 | 0.836107 | 33.33333 | 33.33333 | 1.083608 | 1.083608 | 1.083716 | 1.083716 | 1.071963 | 1.071963 | 1.06 | 1.06 | 1 | 1 | 1.04529 | 1.04529 |
| **2010** | 0.902756 | 0.504683 | 0.557512 | 1.061659 | 1.079514 | 0.076923 | 0.720003 | 33.33333 | 33.33333 | 1.09571 | 1.09571 | 1.09633 | 1.09633 | 1.08487 | 1.08487 | 1.072 | 1.072 | 1 | 1 | 1.04529 | 1.04529 |
| **2009** | 1.332399 | 0.778621 | 0.437613 | 1.105009 | 0.884284 | 0.24359 | 0.841497 | 33.33333 | 33.33333 | 1.107811 | 1.107811 | 1.108945 | 1.108945 | 1.097777 | 1.097777 | 1.084 | 1.084 | 1 | 1 | 1.04529 | 1.04529 |
| **2008** | 2.03937 | 0.967167 | 0.90945 | 1.228176 | 0.770966 | 0.333333 | 0.738151 | 33.33333 | 33.33333 | 1.119912 | 1.119912 | 1.12156 | 1.12156 | 1.110685 | 1.110685 | 1.096 | 1.096 | 1 | 1 | 1.04529 | 1.04529 |
| **2007** | 1.269449 | 1.100108 | 0.953936 | 1.107685 | 0.65561 | 0.346154 | 0.909193 | 33.33333 | 33.33333 | 1.132013 | 1.132013 | 1.134174 | 1.134174 | 1.123592 | 1.123592 | 1.108 | 1.108 | 1 | 1 | 1.04529 | 1.04529 |
| **2006** | 1.44428 | 0.726652 | 1.26007 | 0.988031 | 0.71329 | 0.038462 | 0.755553 | 333.3333 | 333.3333 | 1.144114 | 1.144114 | 1.146789 | 1.146789 | 1.138335 | 1.138335 | 1.12 | 1.12 | 1 | 1 | 1.04529 | 1.04529 |
| **2005** | 1.374867 | 1.135102 | 0.969113 | 1.100073 | 0.769332 | 0.051282 | 0.839868 | 333.3333 | 333.3333 | 1.144114 | 1.144114 | 1.146789 | 1.146789 | 1.140171 | 1.140171 | 1.12 | 1.12 | 1 | 1 | 1.04529 | 1.04529 |
| **2004** | 1.36864 | 1.003636 | 1.0695 | 1.212783 | 0.906976 | 0.051282 | 0.749841 | 333.3333 | 333.3333 | 1.144114 | 1.144114 | 1.146789 | 1.146789 | 1.142007 | 1.142007 | 1.12 | 1.12 | 1 | 1 | 1.04529 | 1.04529 |
| **2003** | 1.442215 | 0.950915 | 1.124913 | 1.30291 | 0.946385 | 6.653846 | 0.743841 | 333.3333 | 333.3333 | 1.144114 | 1.144114 | 1.146789 | 1.146789 | 1.143843 | 1.143843 | 1.12 | 1.12 | 1 | 1 | 1.04529 | 1.04529 |
| **2002** | 1.789517 | 0.977779 | 1.001887 | 0.897386 | 1.007635 | 0.294872 | 0.677945 | 333.3333 | 333.3333 | 1.144114 | 1.144114 | 1.146789 | 1.146789 | 1.143843 | 1.143843 | 1.12 | 1.12 | 1 | 1 | 1.04529 | 1.04529 |
| **Factor Type** | **Activity** | **Activity** | **Activity** | **Activity** | **Activity** | **Activity** | **Activity** | **Sulfur** | **Sulfur** | **PM10** | **PM10** | **PM25** | **PM25** | **NOX** | **NOX** | **CO** | **CO** | **CO2** | **CO2** | **VOC** | **VOC** |
| **PADD** | **1A - East Coast-New England** | **1B - East Coast-Central Atlantic** | **1C - East Coast-Lower Atlantic** | **2 - Midwest** | **3- Gulf Coast** | **4 - Rocky Mountain** | **5 - West Coast** | **Global** | **ECA** | **Global** | **ECA** | **Global** | **ECA** | **Global** | **ECA** | **Global** | **ECA** | **Global** | **ECA** | **Global** | **ECA** |

**Table S7.** Scaling factors for rail by region and pollutant.

| **Year** | **Activity Factor** | **NOx Factor** | **PM10 Factor** | **HC Factor** | **Sulfur Factor** |
| --- | --- | --- | --- | --- | --- |
| 2002 | 0.93195 | 1.499007 | 1.910018 | 1.886327 | 333.3333 |
| 2003 | 1.050021 | 1.463688 | 1.846684 | 1.824553 | 333.3333 |
| 2004 | 0.875097 | 1.428369 | 1.783351 | 1.762779 | 333.3333 |
| 2005 | 0.989998 | 1.39305 | 1.720017 | 1.701005 | 333.3333 |
| 2006 | 1.020092 | 1.357731 | 1.656683 | 1.639231 | 333.3333 |
| 2007 | 1.043662 | 1.30924 | 1.488948 | 1.484553 | 33.33333 |
| 2008 | 0.927397 | 1.292361 | 1.571776 | 1.552846 | 33.33333 |
| 2009 | 0.792296 | 1.256164 | 1.501482 | 1.484878 | 33.33333 |
| 2010 | 0.854178 | 1.219967 | 1.431188 | 1.416911 | 33.33333 |
| 2011 | 0.896248 | 1.18377 | 1.360894 | 1.348943 | 33.33333 |
| 2012 | 0.895387 | 1.147573 | 1.2906 | 1.280976 | 1 |
| 2013 | 0.967643 | 1.111376 | 1.220306 | 1.213008 | 1 |
| 2014 | 1.053949 | 1.083532 | 1.178285 | 1.172358 | 1 |
| 2015 | 1.042326 | 1.027844 | 1.042021 | 1.04065 | 1 |
| 2016 | 1 | 1 | 1 | 1 | 1 |

**Table S8.** Scaling factors for Industrial Biomass Fuel Combustion, Commercial Biomass Fuel Combustion, and Composting Waste Disposal (three subcategories in the nonpt source category).

| **Year** | **Industrial Biomass Fuel Combustion Factor** | **Commercial Biomass Fuel Combustion Factor** | **Composting Waste Disposal Factor** |
| --- | --- | --- | --- |
| 2002 | 0.907163 | 0.824451 | 0.763473 |
| 2003 | 0.885648 | 0.857462 | 0.811633 |
| 2004 | 0.958682 | 0.844359 | 0.859793 |
| 2005 | 0.943082 | 0.843755 | 0.907953 |
| 2006 | 0.956501 | 0.78484 | 0.904593 |
| 2007 | 0.917929 | 0.841555 | 0.901232 |
| 2008 | 0.869669 | 0.87899 | 0.897872 |
| 2009 | 0.765494 | 0.873261 | 0.894511 |
| 2010 | 0.915301 | 0.861463 | 0.891151 |
| 2011 | 0.933864 | 0.831945 | 0.90883 |
| 2012 | 0.949851 | 0.730022 | 0.942434 |
| 2013 | 0.96724 | 0.844081 | 0.991477 |
| 2014 | 0.971127 | 0.91784 | 1 |
| 2015 | 0.958711 | 0.947938 | 1 |
| 2016 | 0.95734 | 0.998513 | 1 |
| 2017 | 1 | 1 | 1 |

**Table S9.** Scaling factors for Residential Wood Combustion emissions.

| **Year** | **Activity Factor** |
| --- | --- |
| 2002 | 0.882963 |
| 2003 | 0.929433 |
| 2004 | 0.952669 |
| 2005 | 0.999722 |
| 2006 | 0.886655 |
| 2007 | 0.979987 |
| 2008 | 1.096655 |
| 2009 | 1.173133 |
| 2010 | 1.258201 |
| 2011 | 1.220349 |
| 2012 | 1.019772 |
| 2013 | 1.330663 |
| 2014 | 1.34668 |
| 2015 | 1.185627 |
| 2016 | 1.032544 |
| 2017 | 1 |

**Table S10. Representative years for spatial surrogates**

| **Source** | **Year(s)** | **12US1 surrogates used** |
| --- | --- | --- |
| afdust | 2002-2017 | 2017-based US |
| ag | 2002-2017 | 2017-based US |
| agfire | 2002 (ag fires = point sources for all other years) | 2017-based US |
| nonptfire | 2002 (all fires = point sources for all other years) | 2017-based US |
| nonpt | 2002-2017 | 2017-based US |
| nonroad (gas and diesel) | 2002-2016 | 2014-based US |
| nonroad (gas and diesel) | 2017 | 2017-based US |
| np_oilgas | 2002-2003 | 2002-based US oil and gas |
| np_oilgas | 2004-2006 | 2005-based US oil and gas |
| np_oilgas | 2007-2009 | 2008-based US oil and gas |
| np_oilgas | 2010-2012 | 2011-based US oil and gas |
| np_oilgas | 2013-2015 | 2014-based US oil and gas |
| np_oilgas | 2016 | 2016-based US oil and gas |
| np_oilgas | 2017 | 2017-based US oil and gas |
| np_solvents | 2002-2017 | 2017-based US |
| onroad (gas and diesel, including California) | 2002-2017 | 2017-based US |
| rail | 2002-2017 | 2014-based US |
| rwc | 2002-2017 | 2017-based US |
